# A Versatile Engineering Platform for the Fabrication of Prosthetic Venous Valves Using Electrospinning

**DOI:** 10.1002/adhm.202504851

**Published:** 2026-02-09

**Authors:** Dario Arcuti, Salma Mansi, Dominic Biebl, Malin Reuter, Maximilian Grab, José Carlos Rodríguez‐Cabello, Petra Mela

**Affiliations:** ^1^ TUM School of Engineering and Design, Department of Mechanical Engineering, Chair of Medical Materials and Implants, Munich Institute of Biomedical Engineering, Technical University of Munich, Germany, Munich Institute of Integrated Materials Energy and Process Engineering Garching Germany; ^2^ Department of Cardiac Surgery, LMU University Hospital Ludwig Maximilian University Munich Munich Germany; ^3^ Deutsches Zentrum für Herz‐Kreislauf‐Forschung (German Centre for Cardiovascular Research) Partner Site Munich Heart Alliance Munich Germany; ^4^ Bioforge Lab (Group For Advanced Materials and Nanobiotechnology) Laboratory For Disruptive Interdisciplinary Science (LaDIS), CIBER‐BBN, Edificio LUCIA Universidad De Valladolid Valladolid Spain

**Keywords:** chronic venous insufficiency, dual electrospinning, elastin‐like recombinamers, small‐diameter heart valves, vascular valves, venous valves

## Abstract

Chronic venous insufficiency caused by valvular incompetence affects millions. Yet, functional prosthetic venous valves (PVVs) remain elusive. Previous concepts failed due to thrombosis and leaflet thickening caused by vascular cell hyperplastic overgrowth. Here, we address these issues by developing a fabrication platform for percutaneous PVVs by fully embedding the stent struts in electrospun fibers that extend luminally to form thin leaflets. This approach offers unique advantages: the electrospun matrix, with spatially controlled porosity, separates the leaflets from hyperproliferating vascular cells and the stent struts from the blood. Its continuity with the leaflets eliminates the need for anchoring points on the stent and associated fixation methods. As a result, stents of any type, cell design or length can be employed, with valve placement possible at any position. We demonstrate these benefits by fabricating bicuspid valves using segments of commercial stents and either single or multiple materials via dual electrospinning, such as thermoplastic polyurethane (TPU) lined with elastin‐like recombinamers (ELRs). The ELR/TPU constructs exhibit minimal platelet adhesion, no hemolysis, and support endothelialization in vitro. Functional evaluation confirms excellent hydrodynamic performance. Finally, the platform's potential for other applications is shown with tricuspid small diameter pediatric heart valves successfully tested according to ISO 8540.

## Graphical abstract

Percutaneous prosthetic venous valves are fabricated by embedding stents in an electrospun matrix that extends luminally to form leaflets. The design shields leaflets from hyperplastic cells, isolates struts from blood contact, and avoids discrete anchoring points. Valves are integrated into stents of different designs, diameters and lengths using single‐ and multi‐material electrospinning, demonstrating good hemodynamics and cellular response.

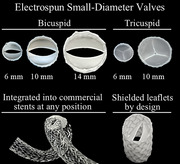

## 1 Introduction

Venous valve dysfunction is a major cause of chronic venous insufficiency (CVI), a prevalent condition that affects up to 40% of adults aged 40–80 years [1]. Valvular incompetence compromises venous return, which, in turn, causes upstream venous hypertension, manifesting with symptoms such as varicose veins, edema, and ulceration [2, 3, 4]. Compression therapy and interventional procedures such as phlebectomy or sclerotherapy are used to relieve the symptoms in mild cases of CVI involving superficial venous networks. For severe deep venous reflux, the therapeutic options aim at restoring valve function by structural repair of the native valve, neovalve construction, or autologous valve transplantation, unfortunately, all with limited long‐term success [5]. Therefore, there is an urgent need for a clinically available functional prosthetic venous valve (PVV). Unfortunately, despite four decades of research, none of the proposed concepts has received approval for clinical use. Device‐related thrombus formation and leaflet rigidity caused by intimal hyperplasia remain major failure mechanisms of venous valve prostheses in vivo, with additional issues such as implant migration and tilting also affecting functionality [6, 7, 8, 9, 10].

The low blood flow and stasis in the venous system create challenging conditions for implants [11] with thrombus formation being observed in in vivo studies of PVVs, regardless of the used material. Thrombi were found in valve cusps made of synthetic materials such as polyether urethane [8], poly(vinyl alcohol) [12], and pellethane [13]. Xeno‐ and allogeneic venous valves have shown high failure rates, primarily due to thrombosis, in animal [14, 15, 16, 17, 18] and clinical studies [19, 20, 21]. Decellularized tissue‐engineered venous valves were tested in sheep, with one valve occluding with a blood clot shortly after implantation and the other failing due to leaflet fusion to the wall [22].

Hyperplastic tissue formation on implanted prosthetic leaflets was also reported as cause of failure, which, as well, occurred regardless of the leaflet material [8, 23, 24, 25, 26, 27]. One strategy to address these limitations involved promoting endothelialization on xenogeneic leaflet materials [28] by in situ pre‐seeding or in vivo cell capture via antibody decoration. Nevertheless, both approaches failed to avoid leaflet thickening in vivo [23]. Notably, in these, as well as in other designs, the leaflets were abutting the vessel wall [8, 23, 24, 25, 26, 27, 29] and, therefore, were accessible to the hyperproliferating vascular cells, triggered by vessel wall injuries that are unavoidable during endovascular prosthesis placement.

Based on these observations, we propose a PVV strategy that combines low‐thrombogenic biomaterials with a design that features a barrier to separate the leaflets from the vessel wall and thus protect them from intimal hyperplasia. We chose solution electrospinning as fabrication technique as it can be applied to a wide range of biomaterials [30, 31], is accessible, scalable [32], has been adapted to increase functionality (e.g., core‐shell fibers [33], porous fibers [34], multifilament threads [35], guided alignment [36, 37]) and still offers unexploited potential for new fabrication schemes. Furthermore, electrospinning enables the production of thin membranes, in contrast to other techniques such as molding, which typically result in thick leaflets [22, 38, 39]. Moriyama et al., [40]. manufactured bicuspid PVVs by electrospinning polyurethane onto a collector carrying a stent. The leaflets were defined on the collector area sticking out of the stent, with the commissures being formed at the ends of two masts protruding from the stent. The collector was either tubular or featured concave surfaces to mimic the native leaflet's shape. In vitro testing revealed high resistance to forward flow for the biomimetic design, while the tubular design performed similarly to a non‐valved scenario in the ankle flexion setup. In both designs, the leaflets were in direct contact with the vein's wall. Other electrospun PVVs were presented by Stiehm et al. [41], who first fabricated bicuspid and tricuspid electrospun sleeves on corresponding collectors, to then release them and fit them inside stents by gluing them to the struts. The authors presented four different leaflet designs and showed that bicuspid valves exhibited, in general, a higher resistance; however they performed better in terms of regurgitation, that could be even higher than 40% for the tricuspid valves. Also, this method did not foresee a barrier between the vessel wall and the leaflets to prevent hyperplasia. The same holds for a recent study [42] where a sleeve was either first electrospun and then fitted abluminally onto a custom‐made stent or was abluminally electrospun on a stent with protruding masts as in Moriyama et al., [40].

In this study, we exploit the versatility of electrospinning to define a new design and fabrication strategy for percutaneous PVVs.

Assisted by additive manufacturing of the collectors, the fabrication scheme consists of multiple electrospinning steps to fully embed a stent into a fibrous matrix that extends luminally to form the valve's thin leaflets within the stent. The presence of the electrospun matrix covering the stent struts and its structural continuity with the leaflets offer several unique advantages: i) integration of a barrier to shield the leaflets with spatially controlled porosity; ii) separation of the stent struts from the bloodstream; iii) no need for additional fixation of the leaflets to the stent by gluing, suturing or welding; iv) no need for specific commissural anchoring points on the stent and, therefore, the possibility to use any stent regardless of the cell design (open or closed). Further benefits of the fabrication platform include the adaptability of the leaflet configurations due to the additively manufactured collectors, and the introduction of dual electrospinning in combination with a spatially controlled material deposition, which enables multi‐material PVVs with optimized mechanical and biological properties, for example, hemocompatibility of the blood‐contacting surfaces.

We demonstrate these advantages by fabricating bi‐ and tricuspid valves using segments of commercially available vascular stents with different cell designs and diameters, as well as custom‐braided magnesium stents. Single‐material and multi‐material PVVs are produced and characterized with respect to their functionality in a mock circulatory system under flow and pressure conditions compatible with the venous circulation.

We also exploit the platform to integrate single or multiple valves into long vascular stents, at any desired position. This could be beneficial for the endovascular treatment of deep venous obstructive disease, another underlying cause of CVI. While venous stents are effective at restoring luminal patency, they do not specifically address the reflux that can either already be present or develop after stenting [43, 44]. By enabling arbitrary placement of valves within long stents, our platform provides a solution that combines recanalization with restoration of unidirectional flow, thereby supporting the development of new therapeutic solutions.

## 2 Results and Discussion

### 2.1 Platform to Fabricate Single‐Material Prosthetic Venous Valves by Solution Electrospinning

**FIGURE 1:**
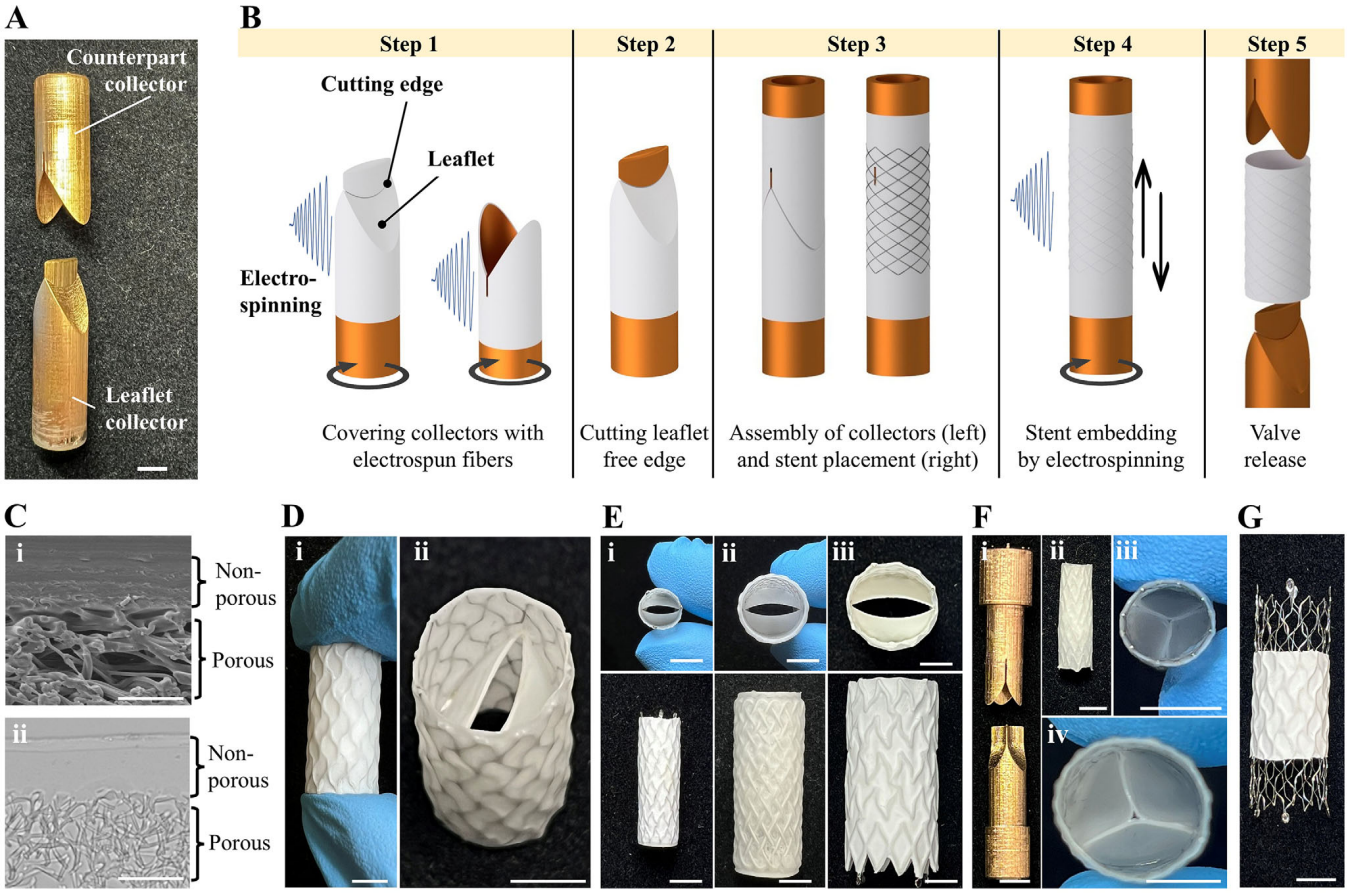
Fabrication of single‐material PVVs. (A) Additively manufactured and gold coated collectors. (B) Schematics of the fabrication process. (C) Cross‐sectional images of an electrospun TPU membrane with non‐porous and porous regions visualized by (i) scanning electron microscopy (SEM) and (ii) bright field microscopy. (D) TPU venous valves as released from the collectors. The membrane covering the stent separates the leaflets from the vessel wall after implantation. (E) The flexibility of the platform is demonstrated with the fabrication of venous valves of different diameters, materials, and stent designs. Specifically: (i) 6 mm, TPU, open‐cell nitinol stent; (ii) 10 mm, PLCL, braided magnesium stent; iii) 14 mm, TPU, open‐cell nitinol stent. (F) Tricuspid valves fabricated with (i) additively manufactured and gold coated collectors and 10 mm segments of (ii, iii) a 6 mm open‐cell nitinol stent and into (iv) a 10 mm closed‐cell nitinol stent. (G) Valve design with partially covered stent. Scale bars: (A, D—F): 5 mm; C: 50 µm.

The fabrication strategy we developed enables the integration of venous valves directly into available vascular stents through an electrospinning process that, concomitantly, also covers the stents by fully embedding the struts. This has the twofold advantage of creating a barrier to prevent cellular ingrowth on the leaflets from the vessel's wall and, at the same time, of providing coverage of the luminal stent surface, thereby isolating the struts from direct blood contact. The fabrication scheme relies on the use of two additively manufactured collectors: the leaflet collector that defines the number, shape, free edges, and normal position of the leaflets, and, the counterpart collector, that complements the leaflet collector to form the membrane covering the stent (Figure 1A). The process steps to fabricate a single‐material bicuspid valve are schematically shown in Figure 1B and demonstrated with TPU. First, TPU fibers were deposited on the leaflet collector (Figure 1B, step 1) by spinning from different angles with respect to the axis of the mandrel on which the collector was mounted, as described in the methods section. By adjusting the electrospinning duration, we obtained a thickness of 146.6 ± 11.4, 162.8 ± 16.2, and 165.4 ± 18.3 µm at the free edge, belly, and base region of the leaflets, respectively. Next, the counterpart collector was also covered with electrospun TPU to create the proximal segment of the covered stent (Figure 1B, step 1). The leaflet's free edges were created by cutting along a predisposed groove on the leaflet collector and by removing the excess material (Figure 1B, step 2). The two collectors were then assembled, creating a form‐fit connection, which enclosed the leaflets. The collector assembly was positioned inside a 20 mm long segment of a self‐expanding closed‐cell nitinol stent (Sinus‐Repo Visual 6F, optimed Medizinische Instrumente, Germany) (Figure 1B, step 3) and TPU was electrospun abluminally, at an increased flow rate and decreased tip‐to‐collector distance, promoting wetter material deposition (Figure 1B, step 4). This was done to ensure adhesion to the luminal layer, resulting in a covered stent segment with struts fully embedded within the electrospun fabric. By further changing the spinning parameters to reduce solvent evaporation in the flight phase of the fiber jet, a non‐porous abluminal membrane can be produced, which will enhance the barrier function against the infiltration of cells from the vessel wall (Figure 1C). Such a non‐porous layer can be positioned abluminally (as described in the methods section), luminally, or interposed anywhere within the thickness of the cover. When created luminally or at an intermediate position, tailored abluminal porosity can facilitate cellular ingrowth, promoting continuity with the vessel wall, which can improve valve anchorage [45, 46]. To finalize the valve, excess material was trimmed and both collectors were removed after overnight air drying to release the valve (Figure 1B, step 5; Figure 1D).

The leaflets of the PVV are formed in a normally open position (Figure 1D(ii)), as in healthy native venous valves, which remain open in the absence of a pressure gradient, such as when the body is in a horizontal position [47, 48], allowing unobstructed blood flow. Furthermore, the leaflets are continuous with the membrane embedding the stent and do not require additional fixation methods such as suturing [17, 22, 23, 38, 39], gluing [41], or welding [29, 42]. This makes the fabrication scheme compatible with any commercially available vascular stent, independently of the diameter and the cell configuration as it does not constrain the stent design by the requirement of specific suturing/anchoring points.

We demonstrated the flexibility of the platform by fabricating TPU PVVs by switching to open‐cell nitinol stent segments of different diameters (i.e., 6 mm Sinus‐SuperFlex‐481 and 14 mm Sinus‐Obliquus, optimed Medizinische Instrumente, Germany) (Figure 1E(i,iii)) as well as by producing a poly(lactide‐*co*‐ε‐caprolactone) (PLCL) valve with a 10 mm self‐braided magnesium stent (Mg 99.9%, 0.25 mm wire diameter, GoodFellow, England) (Figure 1E(ii)). Also, the stent segment length can be adapted, with the minimum length constrained only by the leaflet height. Electrospinning supports a wide material library, therefore, alternative polymers could also be considered, such as poly(ester urethane) urea (PEUU) or supramolecular elastomers, both of which have already demonstrated potential for the fabrication of heart valve substitutes by electrospinning [49, 50].

Furthermore, the platform is not limited to bicuspid configurations. Considering that approximately 10%–15% of native venous valves exhibit a tricuspid leaflet morphology [51], we adapted the collectors’ geometry to fabricate 10 mm and 6 mm tricuspid PVVs (10 mm Sinus‐Repo Visual 6F, optimed Medizinische Instrumente, Germany; 6 mm Epic, Boston Scientific, USA) (Figure 1F). We fabricated the 10 mm valves with thicknesses of 74.3 ± 7.7, 89.1 ± 16.9, and 102.0 ± 25.3 µm at the free edge, belly, and base region of the leaflets, respectively.

Also, the integration of the stent can be varied to leave, for example, the end regions uncovered, which could potentially enhance incorporation into the vessel wall and, consequently, device anchorage (Figure 1G). The verification of this hypothesis requires future in vivo evaluation.

**FIGURE 2:**
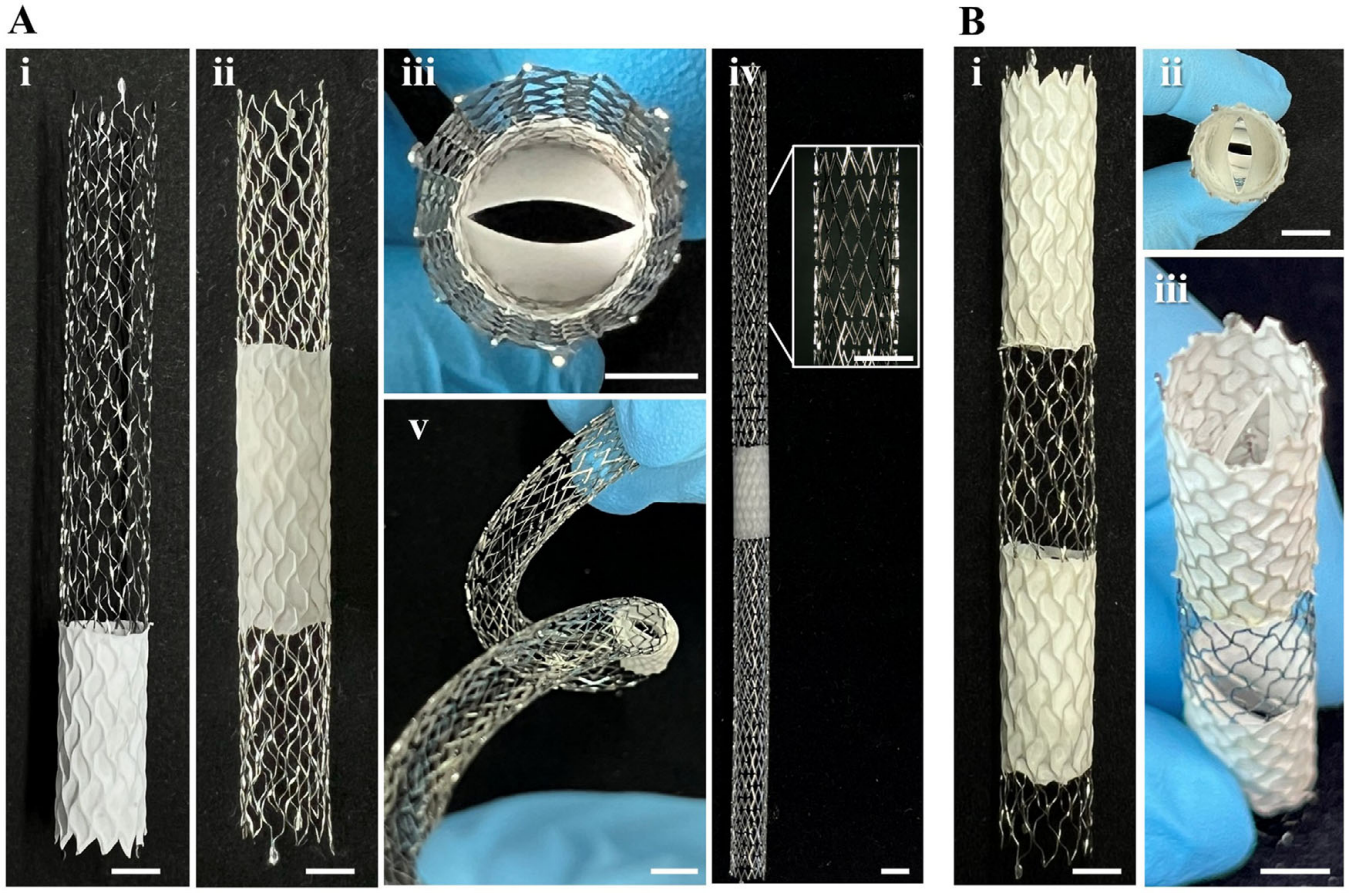
Valves integrated into long stents. (A) Venous valves integrated at the (i) distal and (ii, iii) central region of an 80 mm long closed‐cell vascular stent, as well at the (iv) central region of a 150 mm long open‐cell vascular stent (v) without compromising its flexibility. (B) Valves arranged in a hemodynamically beneficial paired configuration: (i) side view, (ii) top view, and (iii) oblique view of paired valves at 83° in an 80 mm long vascular stent. Scale bars 5 mm.

We further leveraged the versatility of the fabrication scheme and successfully integrated valves into long stents, specifically at the distal and central region of an 80 mm long closed‐cell stent (10 mm Sinus‐Repo Visual 6F, optimed Medizinische Instrumente, Germany) (Figure 2A(i—iii)) as well as at the central region of a 150 mm long open‐cell stent (6 mm Protégé EverFlex, ev3, USA) without compromising the general flexibility of the device (Figure 2A(iv,v)). The capability of equipping long stents with valves at any desired position could be beneficial for the endovascular treatment of obstructive venous lesions, an increasingly performed therapy [52, 53] with stents up to 170 mm in length [52, 54] sometimes combined to treat venous segments measuring up to 283 mm [55]. However, while stenting restores luminal patency with immediate relief of some of the symptoms, it does not address the reflux, which can be already existing or can even develop after stenting, as shown in 22.4% of the treated patients [43]. Augmenting the stent's functionality to ensure unidirectional blood flow could, therefore, open a new therapeutic option.

Native valves are positioned relative to each other at angles of 84.5° in the great saphenous vein (GSV) and 88.3° in the femoral vein (FV), at a distance of 38 mm in the GSV and 46 mm in the FV [56]. Computational studies have demonstrated that arranging paired valves at a 90° relative angle and a separation distance of 40 mm induces a strong helical flow, thereby reducing stagnation regions [57]. Therefore, inspired by these findings, we introduced a sacrificial counterpart collector to integrate valves paired in a hydrodynamically advantageous arrangement (Figure 2B). Although demonstrated here with two, the platform does not restrict the number of valves that can be included.

### 2.2 In Vitro Functional Characterization of Single‐Material Valves

**FIGURE 3:**
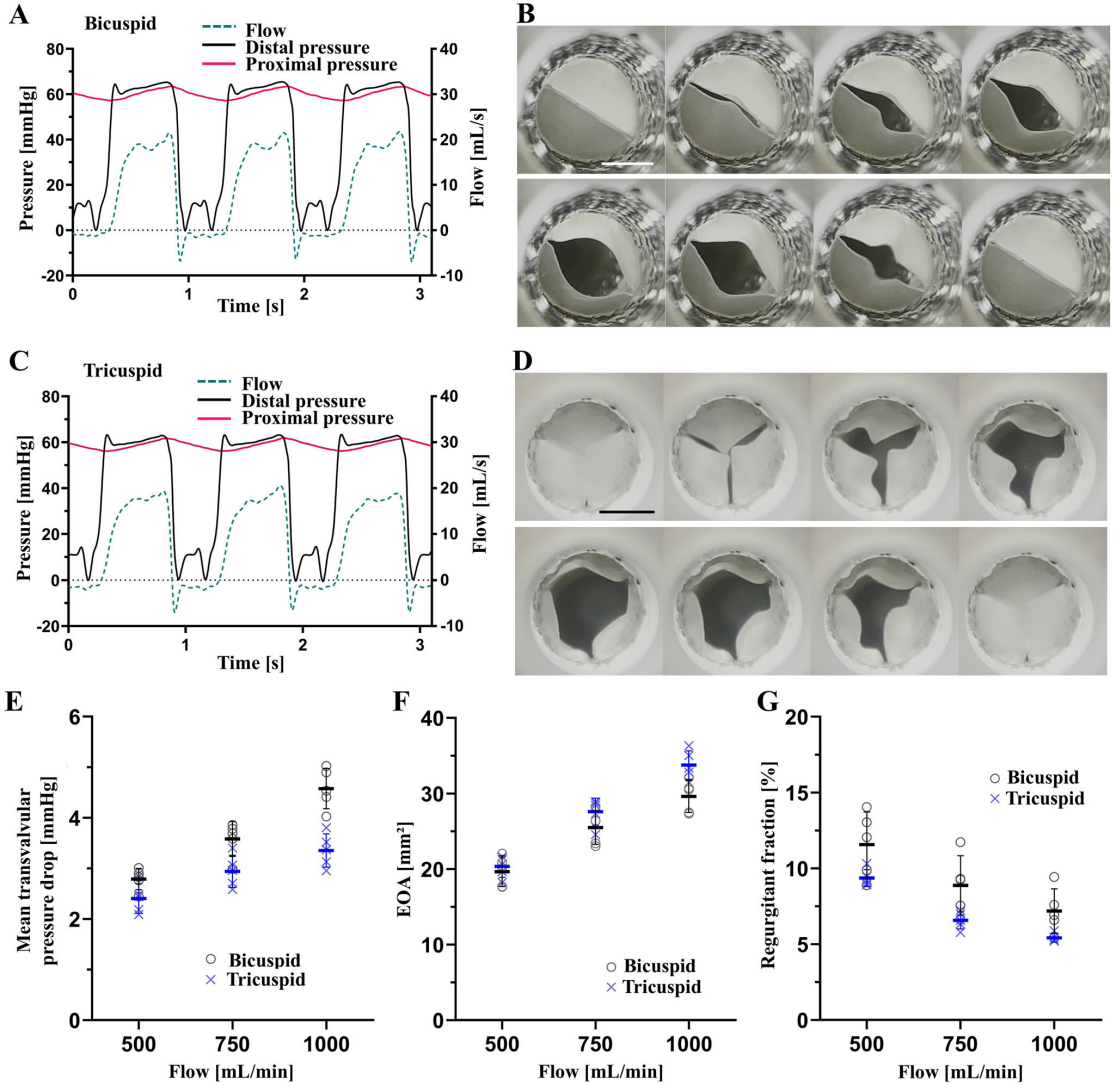
Hydrodynamic evaluation of single‐material TPU valves in a mock circulatory system. (A) Typical pressure and flow profiles and (B) still frames of an opening and closing cycle of a 10 mm bicuspid valve at a flow rate of 500 mL min^−1^. (C) Typical pressure and flow profiles of a 10 mm tricuspid valve at a flow rate of 500 mL min^−1^. (D) Still frames of an opening and closing cycle of a 10 mm tricuspid valve at a flow rate of 750 mL min^−1^. (E) Mean transvalvular pressure drop, (F) effective orifice area, and (G) regurgitant fraction for 10 mm bi‐ and tricuspid valves. Data represented as mean values (n = 5) ± standard deviation and as individual values. Scale bars 5 mm.

We evaluated the 10 mm TPU valves at a proximal pressure of 60 mmHg with flow rates ranging from 500 to 1000 mL min^−1^ in a mock circulatory system (Figure 3A,C). The valves demonstrated unobstructed opening and complete closure (Figure 3B,D) with mean transvalvular pressure drop below 5 mmHg for all tested flow rates (Figure 3E), which is proposed as performance requirement for PVV in the literature [10]. The Effective Orifice Area (EOA) of both leaflet configurations increased with increasing flow rates as expected (Figure 3F), ranging from 25% to 40% relative to the stent's orifice area. In the absence of EOA (%) thresholds for PVVs in the literature and of an ISO standard specific to PVV testing, we referred to performance requirements defined by ISO 5840–3:2021 for transcatheter heart valve substitutes [58]. Specifically, the ISO 5840–3:2021 defines a minimum EOA corresponding to 28%–30% of the stent's orifice area and a regurgitant fraction smaller than 20% [58]. Our results are in line with these requirements also with respect to the regurgitant fraction, which was well below 20% for valves with both leaflet configurations at all flow rates (Figure 3G). It is likely that the regurgitant fraction measured in vitro is affected by the porosity of the leaflets and, therefore, overestimated with respect to the in vivo performance, as it is expected that the electrospun leaflets will be infiltrated by blood and thereby sealed instantaneously upon implantation. This assumption is supported by in vivo measurements performed by Uiterwijk et al., who measured low regurgitation on echocardiography immediately after heart valve implantation, despite elevated regurgitation values measured in vitro [50]. Further reduction of the regurgitant fraction could be achieved by decreasing the closing volume through leaflet design optimization, which can be readily implemented by means of additively manufactured collectors.

Additionally, we evaluated the hydrodynamic performance of a 6 mm bicuspid TPU valve at flow rates of 150 to 500 mL min^−1^, which showed unobstructed opening and complete closure (Figure S1A,B). As expected for very small diameters, the valves exhibited higher transvalvular pressure drops (Figure S1C), which could be addressed through optimization of leaflet geometry and material selection.

Inspired by the off‐label use of the Medtronic's Melody pulmonary heart valve, which has been surgically implanted at the aortic position in pediatric patients [59], we assessed the hemodynamic behavior of the tricuspid 10 mm valve under neonatal (65/45 mmHg, 150 bpm) and infant (88/50 mmHg, 120 bpm) aortic conditions, according to ISO 5840–1:2021 [60]. The valves showed uniform leaflet mobility and excellent hydrodynamic behavior (Figure S2). The ISO 5840 does not specify minimum performance requirements for pediatric valves, that is, valves with small diameters. Therefore, we referred to the ISO 5840–2:2021 criteria for surgically implanted adult valves. This indicates a minimum EOA corresponding to 28%–30% of the annular area and a regurgitant fraction smaller than 10% [61]. The tested valves satisfied both criteria, with an EOA (Figure S2F) ranging from 40% to 53% relative to the stent's orifice area, and the regurgitant fraction remaining below 9% for all tested flow conditions (Figure S2G). This opens the possibility to have pediatric valves with diameters not yet present on the market, where the smallest available diameters are 12 and 15 mm for biological (Contegra, Medtronic) and mechanical (Masters HP, Abbot) heart valves, respectively.

### 2.3 Multi‐Material Layered Constructs by Dual Solution Electrospinning

**FIGURE 4:**
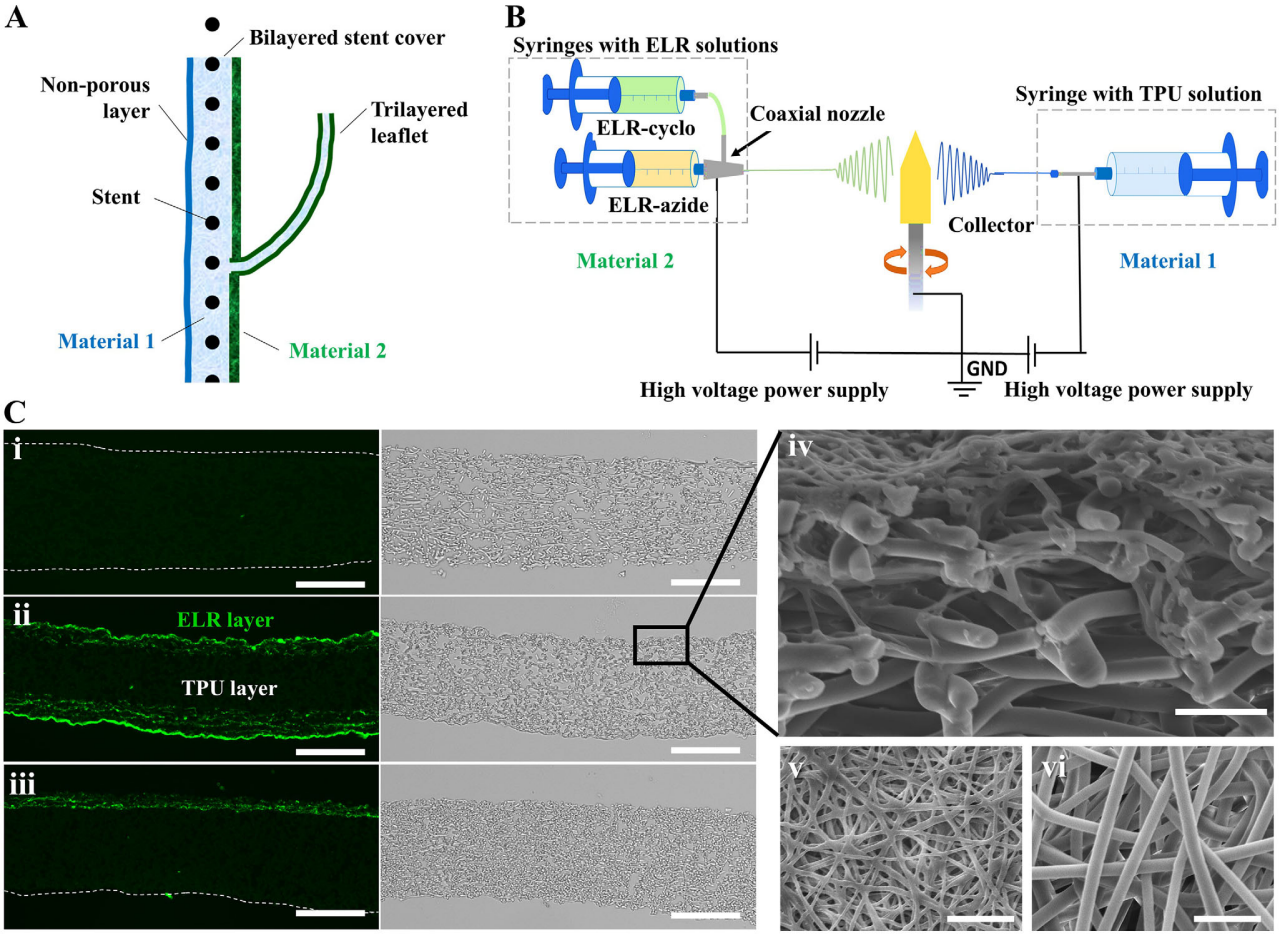
Fabrication of multi‐material constructs. (A) Schematic representation of the PVV design foreseeing two distinct materials for the core layer and the blood contacting surfaces, which results in bi‐ and trilayered regions. (B) Schematic representation of the electrospinning arrangement to produce layered constructs from two distinct materials, here depicted as dual electrospinning of ELR and TPU. (C) Fluorescence and brightfield images of the cross‐sectional microstructure of (i) electrospun TPU, (ii) trilayered ELR/TPU, and (iii) bilayered ELR/TPU constructs. ELR fibers were visualized by taking advantage of their autofluorescence. (iv) SEM image showing the ELR layer on top of the TPU layer. (v) ELR and (vi) TPU fibers are distinguishable by their characteristic diameters and morphologies. Scale bars: (C) (i—iii): 100 µm, (iv—vi): 10 µm.

Next, we advanced the fabrication platform to enable multi‐material electrospinning in order to confer specific properties to the valve, such as mechanical strength provided by material 1 and hemocompatibility provided by material 2 to all blood‐contacting surfaces (Figure 4A), considering that thrombus formation remains a major limitation of previous PVV approaches. This required the realization of bi‐ and trilayered constructs, for the wall and leaflets, respectively. To this end, two polymer solutions were alternatingly electrospun from distinct nozzles onto a shared collector to obtain layered constructs. During the transition phase from one material to the other, we applied dual electrospinning, in which both solutions were electrospun concomitantly to create mixed fiber entanglement and thus adhesion between the layers.

Because of the excellent hemodynamic performance shown by the single‐material valves, we chose to use TPU as mechanically defining material and ELRs for the blood contacting surfaces. Inspired by native elastin, ELRs are characterized by repetitive sequences of the Val‐Pro‐Gly‐Xaa‐Gly pentapeptide motif found in natural elastin and can be further engineered through the integration of bioactive sequences and chemical groups to enable a catalyst‐free click reaction [62]. ELRs are promising biomaterials for cardiovascular applications, due to their minimal thrombogenicity [63, 64, 65] and ability to support endothelial layer formation [63, 66]. We previously manufactured ELR scaffolds by various methods including electrospinning [67], molding [63, 66], dip‐coating [68], and salt leaching/gas foaming [69].

The fabrication strategy required two independent electrospinning systems, one of which was predisposed for coaxial electrospinning to enable the click reaction between the two ELR components functionalized with cyclooctyne and azide groups (Figure 4B). The resulting bi‐ and trilayered configuration of ELR/TPU constructs was confirmed by visualization of cross‐sectional cuts using SEM and fluorescence microscopy, taking advantage of ELRs’ autofluorescence (Figure 4C).

In the next step of this study, the biological response of the layered ELR/TPU constructs was evaluated in vitro by platelet adhesion and hemolysis assays, as well as by assessing endothelization and smooth muscle cell infiltration.

**FIGURE 5:**
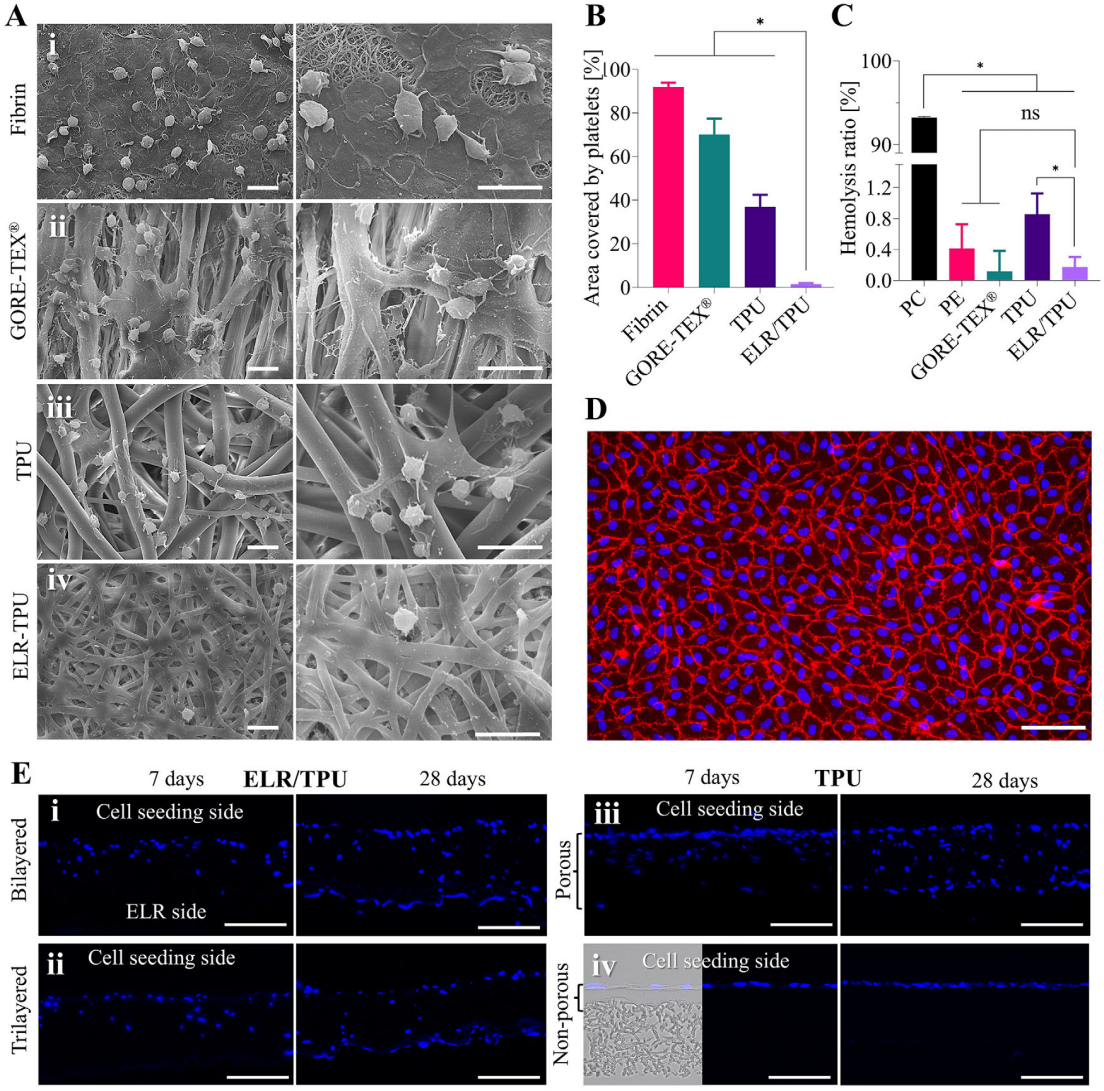
Characterization of layered ELR/TPU constructs. (A) SEM images of platelet adhesion on (i) fibrin, (ii) GORE‐TEX, (iii) TPU, and iv) ELR/TPU constructs. Zoomed‐in views show platelets in different states of activation: fully activated platelets on GORE‐TEX, fibrin, and TPU, and minimally activated platelets on ELR. (B) Quantification of platelet coverage showing significantly lower adhesion on ELR surfaces compared to GORE‐TEX, TPU, and fibrin. Values represent mean ± standard deviation as obtained from n = 5 independent samples. (C) Hemolysis induced by ELR/TPU constructs was comparable to GORE‐TEX and polyethylene (PE) control, and lower than that induced by TPU. Whole blood diluted in deionized water served as the positive control (PC). Values represent mean ± standard deviation as obtained from n = 5 independent samples. (D) Fluorescence image of the endothelialized ELR surface after 72 h of static culture on ELR/TPU constructs, showing CD31 (red) staining and DAPI nuclear counterstain (blue). (E) Fluorescence images of cross‐sectional cuts used to assess SMC infiltration with DAPI nuclear counterstain (blue). Infiltrating cells were visible at day 7 and 28 of culture in (i) bilayered and (ii) trilayered ELR/TPU constructs as well as in (iii) porous TPU, while (iv) TPU samples featuring a non‐porous region prevented SMC infiltration. Asterisks (*) mark statistically significant differences based on a *p*‐value of 0.05, “ns” indicates non‐significant differences. Scale bars: (A): 5 µm, (D,E): 100 µm.

Platelets on the GORE‐TEX and fibrin samples were found in a highly activated state, evident from their fully spread morphology and extensive filopodia and lamellipodia formation (Figure 5A(i,ii)). On TPU samples the platelets appeared to be also activated, although to a lower extent (Figure 5A(iii)), while on the ELR samples (Figure 5A(iv)), only a low number of platelets were visible with minimal pseudopodia, indicating a low activation state [70]. Moreover, quantification of platelet coverage showed significantly lower adhesion on ELR surfaces compared to GORE‐TEX, TPU, and fibrin (Figure 5B).

A favorable result for the ELR/TPU samples was also given by the hemolysis assay showing a hemolysis ratio below 2% (Figure 5C), which is commonly considered as the threshold to classify a material as non‐hemolytic [71]. Notably, the ELR/TPU constructs exhibited significantly lower hemolysis compared to the pure TPU fabric. However, it must be considered that both assays were conducted in vitro under static conditions. Dynamic setups, such as a Chandler loop, can provide a better understanding of material–blood interactions under more physiological conditions.

Successful formation of a confluent endothelial cell layer on the ELR surfaces was visualized after 24 and 72 h of static culture on ELR/TPU constructs, while an incomplete layer was observed on the TPU membranes, as shown by CD31 immunostaining and DAPI counterstaining (Figure 5D and Figure S3). These results agree with previous studies indicating that RGD‐functionalized ELRs, such as the one used in this study, support endothelialization [39, 63, 66, 67, 68]. In this work, we used a bioactive ELR bearing an RGD cell adhesive motif, however, endothelial‐specific sequences (e.g., REDV) can be introduced in ELRs [63] as well as endothelial progenitor cell capturing sequences to facilitate fall‐out endothelialization [72]. Replacing the RGD could further reduce the thrombotic risk as it binds platelet receptors [73].

Cell infiltration in electrospun scaffolds is hindered by the typically dense fiber architecture resulting in small pore sizes [74]. Increasing the diameter of the fibers results in increased pore size [75], therefore, cell infiltration can be steered by controlling the electrospinning process. Alternatively, a non‐porous barrier to halt cell migration can be created by wet electrospinning, independently of the fiber size, as shown in our experiments (Figure 5E and Figure S4), where infiltration of primary human umbilical vein smooth muscle cells (HUVSMCs) into bi‐ and trilayered TPU/ELR constructs, as well as single‐material porous TPU samples, was visible at days 7 and 28 of cultivation (Figure 5E(i–iii) and Figure S4A,B(i—iii)), whereas the TPU samples featuring a non‐porous region effectively prevented it (Figure 5E(iv) and Figure S4A,B(iv)). These experiments also highlight the potential of the electrospun layered constructs as scaffolds for tissue engineering applications. We have shown the possibility of using PLCL as a material for the fabrication of the PVVs (Figure 1E(ii)) and a wide range of biodegradable materials could be readily processed. For example, we could exploit the continuously expanding library of ELRs with tailored functionalities such as protease‐sensitive sequences [76], or silk fibroin, known for its tunable degradation profile and ability to support endothelial cell adhesion [77]. Moreover, a core layer of PCL could be complemented with surface layers functionalized with nitric oxide precursors and heparin to modulate thrombogenicity [78].

Next, the mechanical properties of the electrospun TPU and the trilayered ELR/TPU constructs were determined by biaxial tensile testing. The resulting stress–strain curves (Figure S5A) showed the non‐linear, hyperelastic response typically observed for TPU [79, 80]. Tensile moduli for both constructs were calculated from the slope of the stress–strain curves in the 0%–5% and 20%–40% strain ranges (Figure S5B). All samples exhibited slight anisotropy, which likely arose from fiber alignment caused by favored fiber deposition parallel to the mandrel axis during electrospinning onto a slowly rotating cylindrical collector. Liu et al. reported that a cylindrical collector induces a non‐uniform electric field that changes the bending cone from circular to elliptical, thereby biasing fiber deposition toward the mandrel axis [81]. Similar collector‐induced fiber alignment and associated anisotropy have been reported in electrospun tubular constructs [50]. The ELR/TPU constructs exhibited slightly higher stiffness in the low‑strain range compared to the TPU, which can be attributed to the stiffness of elastin in its dry state [82].

The trilayered ELR/TPU constructs exhibited a burst strength of 1367 ± 87 mmHg, which is within the range reported for the human saphenous vein (1600 ± 877 mmHg) [83]. Another important aspect to evaluate is the mechanical integrity of the layered constructs under flow conditions. Therefore, we proceeded to expose bilayered ELR/TPU constructs for 1 h to a flow‐induced shear stress of 30 dyn cm^−2^, which is much higher than those expected in vivo [84]. When examined by SEM, no delamination was observed (Figure S6A–C), confirming the suitability of the dual electrospinning to achieve interlayer adhesion.

### 2.4 Multi‐Material Valve Fabrication by Dual Electrospinning

**FIGURE 6:**
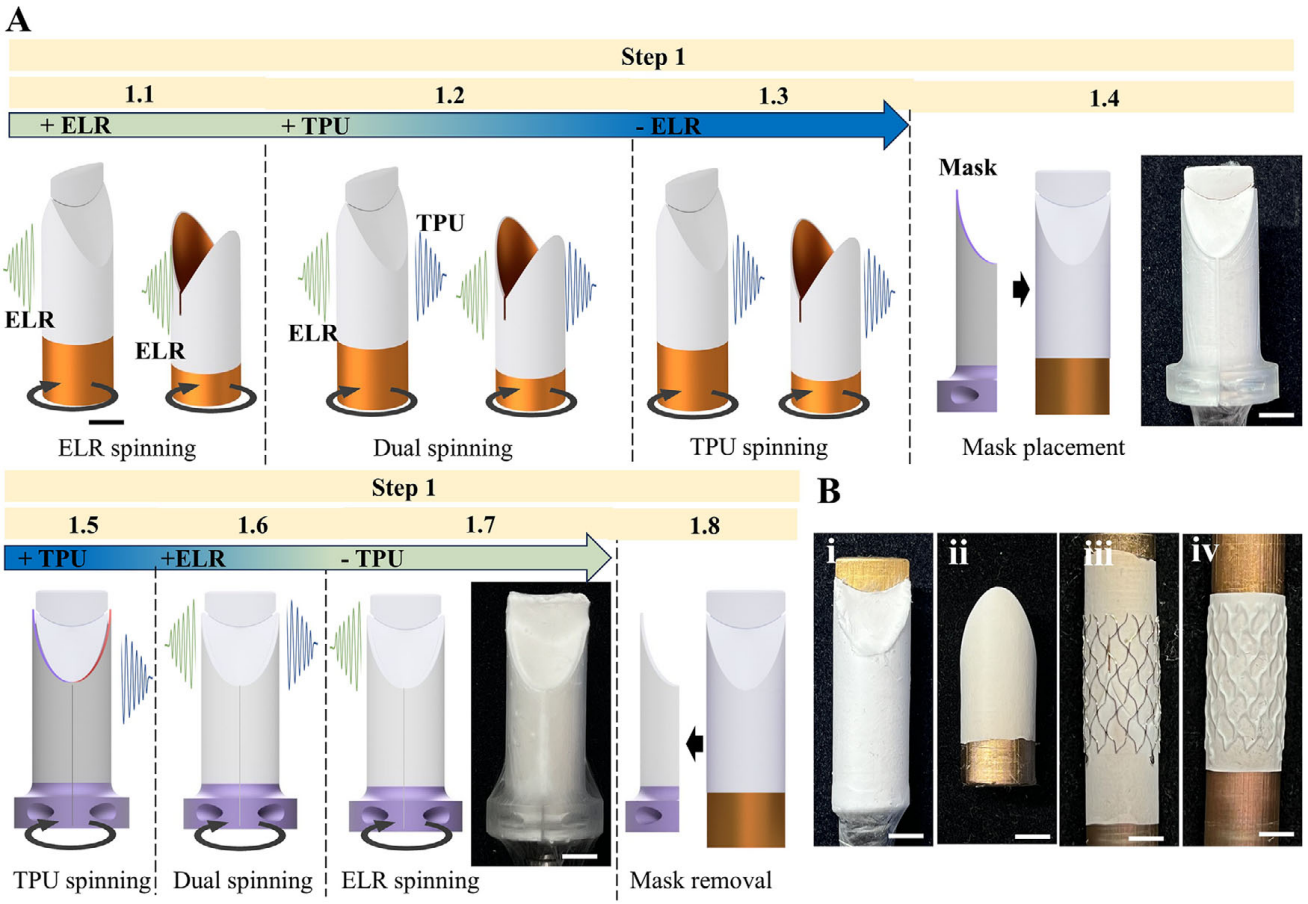
Fabrication strategy for ELR/TPU valves. (A) Schematic of modified fabrication step 1: (1.1) ELR fiber deposition onto the collectors; (1.2) dual electrospinning of ELR and TPU; (1.3) deposition of pure TPU fibers; (1.4) placement of an additively manufactured mask; (1.5) selective TPU fiber deposition onto the leaflet region; (1.6) second dual electrospinning of ELR and TPU; (1.7) electrospinning of pure ELR to form the parietal surface of the leaflet; and (1.8) mask removal. (B) Images of the fabrication steps 2–5: (i) collector after mask removal with cut leaflet free edge, (ii) counterpart collector covered with bilayered ELR/TPU electrospun fabric, (iii) assembly of both collectors and placement of a vascular stent segment, and (iv) covered stent segment with struts fully embedded within the electrospun fabric. Scale bars 5 mm.

Next, we advanced the fabrication concept shown in Figure 1 to manufacture a multi‐material valve consisting of a bilayered ELR/TPU covered stent segment and trilayered leaflets, achieved by spatially selective material deposition through sequential spinning steps and masking of specific collector regions. This required the modification of valve fabrication step 1 in the protocol used for single‐material valve fabrication (Figure 1B), while steps 2–5 remained unchanged. Specifically, the deposition of the materials on the collectors prior to their assembly required eight consecutive steps, instead of just one. To maintain the correspondence to the schematic shown in Figure 1B, we indicate these steps as collectively belonging to step 1 (Figure 6A). Steps 1.1–1.3 were identical for both collectors and will be detailed only for the leaflet collector, which additionally requires steps 1.4–1.8.

The process began with the coaxial electrospinning of click‐ELR components onto the rotating leaflet collector, forming the initial ELR fiber layer that constitutes the luminal surface of the valve leaflets and the distal portion of the covered stent (the proximal portion of the stent is formed in the same way on the counterpart collector, in a separate process) (Figure 6A, step 1.1). This was followed without interruption by dual electrospinning of click‐ELR and TPU to promote inter‐fiber entanglement of the two materials and, thus, interfacial adhesion (Figure 6A, step 1.2). Next, the electrospinning of ELRs was discontinued to deposit a layer of pure TPU fibers (i.e., the mechanically defining core) (Figure 6A, step 1.3). At this stage, the process was temporarily paused to place an additively manufactured mask onto the collector (Figure 6A, step 1.4), leaving only the leaflet region exposed to form the ELR parietal side of the leaflets by transitioning from TPU to ELR through dual spinning (Figure 6A, steps 1.5–1.7). At this stage, the mask was removed (Figure 6A, step 1.8) and the free edges of the leaflets were defined by cutting the excess material (Figure 1A, step 2). Next, the collectors are ready (Figure 6B(i,ii)) to be assembled and placed inside a stent segment (Figure 1A, step 3; Figure 6B(iii)) to then proceed with the final TPU electrospinning (Figure 1A, step 4), removal of excess material (Figure 6B(iv)) and the valve release (Figure 1A, step 5).

With the electrospinning parameters we describe in the methods section, we obtained a leaflet thickness of 88.5 ± 19.1, 121.0 ± 33.8, and 118.5 ± 32.4 µm at the free edge, belly, and base region, respectively.

### 2.5 In Vitro Functional Characterization of Multi‐Material Valves

**FIGURE 7:**
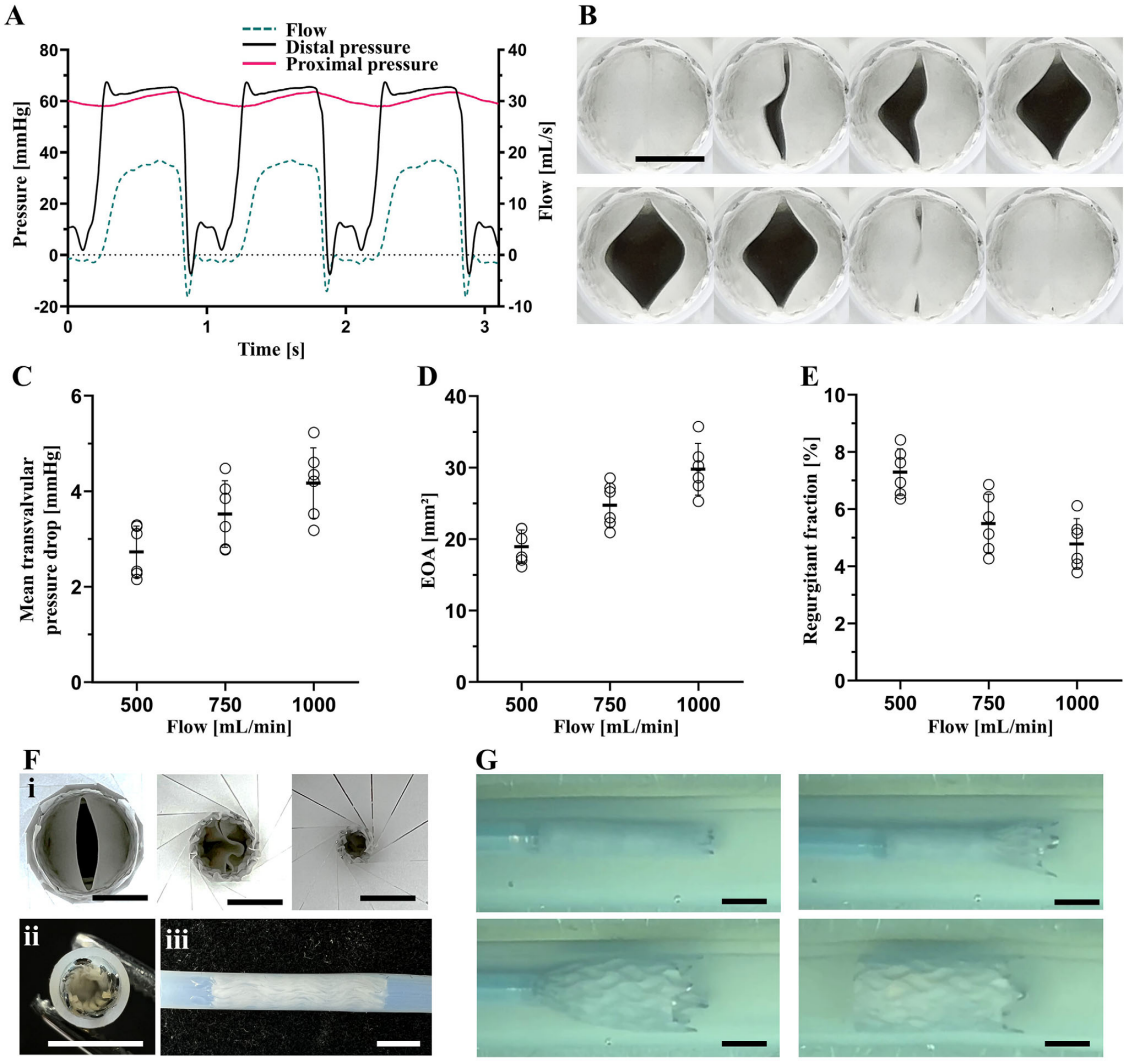
In vitro functional characterization of 10 mm ELR/TPU valves. (A) Typical pressure and flow profiles at a flow rate of 500 mL min^−1^. (B) Still frames of an opening and closing cycle at a flow rate of 1000 mL min^−1^. (C) Mean transvalvular pressure drop, (D) EOA, (E) and regurgitant fraction. Data represented as mean values (n = 6) ± standard deviation and as individual values. (F) Images showing (i) crimping from 10 to 2.8 mm (8.4 Fr); (ii) cross‐sectional and (iii) side view of crimped valve loaded into 12 Fr delivery tube. (G) Still frames from a simulated delivery procedure into a 10 mm silicone tube. Scale bars 5 mm.

The in vitro hemodynamic performance of the ELR/TPU venous valves was assessed under the same flow and pressure conditions previously established for the single‐material valves. Also, the ELR/TPU valves demonstrated unobstructed opening and complete closure (Figure 7A,B) and exhibited favorable fluid dynamics. The mean transvalvular pressure drops were well below 5 mmHg even at the highest flow rate (Figure 7C), thereby meeting the performance requirement [10]. The EOA of the tested valves also increased with flow rate (Figure 7D), ranging from 24% to 38% relative to the stent's lumen. Notably, the regurgitant fraction of the valves remained below 9% across all tested flow rates (Figure 7E) satisfying the ISO 5840‑3:2021 standard for transcatheter prosthetic heart valves, which requires a regurgitant fraction smaller than 20% [58].

Next, to evaluate the possibility for transcatheter implantation, we crimped a 10 mm ELR/TPU valve to a diameter of 2.8 mm (Figure 7F(i)) and loaded it inside a mock delivery system (Figure 7F(ii,iii)) consisting of a PTFE tube with an inner diameter of 3.2 mm and an outer diameter of 4 mm (12 Fr). The valve was then released inside a 10 mm silicone tube submerged in water at 37°C (Figure 7G), where it self‐expanded unobstructed to its original diameter. Subsequent hemodynamic testing of the valve under the previously applied conditions showed no change in performance in comparison with that before the implantation test, highlighting the stability of the construct and its potential for clinical translation. When examined by SEM, the valve surface showed no apparent microstructural damage after crimping and simulated delivery (Figure S7A,C,E,G) and the surface morphology was comparable to that of valves that were never crimped (Figure S7B,D,F,H). In addition, 10 mm TPU valves were functionally evaluated as fabricated and after they were crimped, deployed to simulate delivery and, subsequently, subjected to 500 000 cycles in a custom‐made durability tester at 10 Hz and 60 mmHg of differential pressure across the closed valve. No statistically significant differences were observed in mean transvalvular pressure drop, effective orifice area, or regurgitant fraction (Figure S8). The choice of 500 000 cycles follows the recommendation by Sathe et al., [85]. for a proof‐of‐concept evaluation of laboratory‐fabricated valves. However, a durability assessment needs to be performed to confirm long‐term mechanical robustness. As TPU is the mechanically defining material in the ELR/TPU constructs, we expect the fatigue response of the ELR/TPU valves to be similar to that of TPU valves.

Notably, the delivery tube used in this study has a smaller diameter than the proposed catheter size for PVVs of 16 Fr in literature [10], as well as the 13 Fr system required for a recently introduced endovascular PVV approach [86]. This reduction offers potential clinical advantages, including a lower risk of catheter induced thrombosis, and conforms to literature recommendations for vein‐to‐catheter ratios [87]. Although demonstrated only in a preliminary setup, the findings suggested that the use of commercially available stent segments enables integration with existing delivery platforms, eliminating the need for dedicated device development.

## 3 Conclusion

We presented a versatile engineering platform for small‐diameter, multi‐material prosthetic valves suitable for venous valve replacement. The electrospinning‐based fabrication approach is not limited to the specific materials used in this study and can be readily applied to the practically unlimited library of biomaterials processable by this technique, and specialties such as core–shell electrospinning may further augment the platform's functionality by enabling features such as localized and timed drug delivery. We demonstrated compatibility with a wide range of commercially available vascular stents; however, the approach is not restricted to any specific stent architecture or size, offering broad versatility for future clinical applications. Notably, the platform enabled the integration of multiple valves into long stents, offering a potential solution for simultaneously addressing venous obstruction and reflux within a single device. Although presented for small‐diameter venous valves, the platform can be exploited for other therapeutic solutions as demonstrated with the fabrication of tricuspid heart valve prostheses for pediatric patients, for whom current options remain limited.

## 4 Experimental Section

### 4.1 Electrospinning

Electrospinning was performed on an in‐house developed setup. Briefly, the system consisted of two independent screw‐driven linear stages for the *X* direction and one stage for the *Y* direction. The rotating mandrel was mounted on the Y‐axis stage, while two syringe pumps (New Era Pump Systems, USA; Fisher Scientific, Germany) were mounted on independent X‐axis stages and arranged perpendicularly to the mandrel's axis, on opposite sides. The distance between each syringe needle and the mandrel was adjusted by translating the corresponding X‐axis stage. By translating the Y‐axis stage, the axial position of the mandrel relative to the syringe needles was adjusted, allowing control over which region of the mandrel the needles were directed at. Additionally, the relative angle between the syringe needles and the mandrel's axis was adjusted during fabrication by rotating the mandrel with a rotary stage (YXRA100, Beijing Yixuan, China) mounted on the Y‐axis stage. Two high‐voltage power supplies (PHYWE Systeme, Germany) applied independent positive potentials to the syringe needles, while the metal mandrel was grounded.

To manufacture valves made of a single material, only one syringe pump was used. The collectors were designed using Autodesk Inventor 2024 Professional (Autodesk, USA), additively manufactured by vat photopolymerization (Clear resin, Form3, Formlabs, USA) and postprocessed according to the manufacturer's instructions. The collectors were then coated with a 40 nm gold layer by sputtering (SCD 005 Sputter‐Coater, BAL‐TEC, Germany) and were mounted on the grounded mandrel via their inner thread. A conductive connection between the collector and the mandrel was achieved by plastic conductive carbon cement (LEIT‐C‐PAST, Plano, Germany).

### 4.2 Electrospinning Solutions

Separate solutions of medical grade polycarbonate‐based aliphatic thermoplastic polyurethane (TPU; Carbothane PC‐3555D, LUBRIZOL Life Sciences, USA) and poly(lactide*‐co‐*ε‐caprolactone) (PLCL; PURASORB PLC 7015, Corbion, Netherlands) were prepared by dissolving each polymer in 1,1,1,3,3,3‐hexafluoro‐2‐propanol (Carbolution Chemicals, Germany) at concentrations of 0.075 and 0.12 g mL^−^^1^, respectively, and stirring at 300 rpm overnight at room temperature (MR 3001, Heidolph, Germany). For the blood‐contacting surfaces, elastin‐like recombinamers (ELRs) chemically modified for catalyst‐free click chemistry (Technical Proteins Nanobiotechnology, Spain) were used. Specifically, we chose a bioactive ELR containing an RGD sequence and azide groups (ELR‐RGD), and a structural ELR modified with cyclo‐octyne groups (ELR‐VKVx24) [62]. Each ELR component was dissolved at a concentration of 150 mg mL^−1^ in a 1:1 mixture of phosphate‐buffered saline (PBS) (Gibco, Germany) and ethanol (EMSURE, Merck, Germany) [67]. After stirring at 300 rpm at room temperature for 1 h, the solutions were loaded into 1 mL syringes (B.Braun, Germany) for immediate electrospinning.

### 4.3 Single‐Material PVV Fabrication

The polymer solution was electrospun with one syringe pump through an 18G needle onto the leaflet collector at a flow rate of 2.5 mL h^−^^1^ (TPU) or 1.8 mL h^−^^1^ (PLCL), with a voltage difference of 10 kV (TPU) or 12 kV (PLCL) and a tip‐to‐collector distance of 12 cm. The electrospinning process consisted of first covering the leaflet collector, then the counterpart collector, both over a total length of 20 mm, to then assemble them together, position them into the stent and spin the abluminal coverage as final step (Figure 1B). Initially, with the leaflet collector stationary and positioned at a 90° angle relative to the syringe needle, each leaflet was covered twice for its entire height (10 mm), each time for 30 s. This was followed by 2 min of fiber deposition with the collector rotating at 50 rpm, maintaining the 90° alignment. Subsequently, the collector was positioned at a 45° angle relative to the needle, and each leaflet was again coated twice for 30 s without rotation. Next, the collector rotated at 50 rpm for an additional 1 min of electrospinning. To additionally cover 10 mm of the leaflet collector's shaft, the collector was repositioned at a 90° angle to the needle, translated 10 mm, and electrospinning was performed in this position for the last 2 min. Thereafter, the leaflets' free edges were created by cutting along the predisposed groove on the leaflet collector with a scalpel and by removing the excess material. In a separate fabrication process, the counterpart collector was covered by electrospinning the polymer solution for 3 min (TPU: 18G, 2.5 mL h^−1^, 10 kV, 12 cm, 50 rpm, 90°; PCLC: 18G, 1.8 mL h^−1^, 12 kV, 12 cm, 50 rpm, 90°). Bridging fibers were removed manually with a scalpel before the two collectors were assembled and inserted into the nitinol stent. Valves were fabricated using either vascular stent segments of variable length or entire long stents. We used closed‐cell nitinol stents (Sinus‐Repo Visual 6F, optimed Medizinische Instrumente, Germany), open‐cell nitinol stents (Sinus‐SuperFlex‐481 and Sinus‐Obliquus, optimed Medizinische Instrumente, Germany; Protégé EverFlex, ev3, USA) and a custom‐made braided magnesium stent (Mg 99.9%, GoodFellow, England). The polymer solution was electrospun on the collector‐stent assembly for 5 min at a distance of 8.5 cm, followed by 7 min at a distance of 12 cm (TPU: 18G, 3.5 mL h^−^^1^, 10 kV, 50 rpm, 90°; PLCL: 18G, 3.0 mL h^−^^1^, 12.6 kV, 50 rpm), resulting in complete stent embedding. During this process, the mandrel was moved in an alternating translational motion at a speed of 10 mm s^−1^, over a length of 40 mm. To integrate an abluminal dense layer, the polymer solution was subsequently wet‐spun onto the collector‐stent assembly for 5 min by reducing the tip‐to‐collector distance to 8 cm (TPU: 18G, 3.5 mL h^−1^, 10 kV, 50 rpm, 10 mm s^−1^, 90°; PLCL: 18G, 3.0 mL h^−^^1^, 12.6 kV, 50 rpm).

### 4.4 ELR/TPU PVV Fabrication

Two syringe pumps arranged on opposite sides of the mandrel were used, one loaded with a TPU solution, the other with the two ELR click components. Electrospinning enabled regional selective deposition of materials, achieved either through sequential spinning steps or by masking specific regions to exclude them from fiber deposition. Initially, the leaflet collector was coated over a length of 20 mm with a layered ELR/TPU construct. First, ELR fibers were deposited by coaxial electrospinning of the two click chemistry ELR components onto the rotating leaflet collector positioned at a 90° angle relative to the coaxial needle (22G/17G) for 3 min at a flow rate of 0.37 mL h^−1^, with a voltage difference of 10 kV, a tip‐to‐collector distance of 14 cm, and a rotation speed of 50 rpm. Electrospinning of TPU was then started (18G, 1.7 mL h^−1^, 12 kV, 14 cm) and carried out for 5 min without stopping the electrospinning of ELR (dual electrospinning). Subsequently, the electrospinning of ELRs was discontinued and only TPU fibers were continuously deposited for another 5 min (18G, 2.5 mL h^−1^, 10 kV, 12 cm, 50 rpm). Afterward, the rotation was halted, and the TPU fiber jet was focused on each leaflet region for 60 s. Next, the spinning was shortly interrupted and a mask was placed on the leaflet collector leaving only the leaflet regions exposed to the fiber jet. The mask was additively manufactured by vat photopolymerization (Clear resin, Form3, Formlabs, USA) and consisted of two identical halves that were attached laterally to the leaflet collector (Figure 6A) and connected at the bottom with two pins to secure them in place. Electrospinning was then continued by focusing the TPU fiber jet (18G, 2.5 mL h^−1^, 10 kV, 12 cm) onto each leaflet side for 2 min in stationary conditions, followed by TPU spinning onto the rotating collector (50 rpm) for 5 min. Without interrupting the process, dual electrospinning was performed with click‐ELR solution (22G/17G, 0.37 mL h^−1^, 10 kV, 14 cm) and TPU solution (18G, 1.7 mL h^−1^, 12 kV, 14 cm) together for 5 min onto the rotating collector (50 rpm). Finally, an ELR layer was deposited by electrospinning of click‐ELR solution (22G/17G, 0.37 mL h^−1^, 10 kV, 14 cm, 50 rpm). As a result, the leaflets consisted of a trilayered ELR/TPU construct, while the stent cover consisted of a bilayered ELR/TPU construct. The mask was carefully removed and the leaflets' free edges were created by cutting along the preformed groove on the leaflet collector with a scalpel and removing the remaining excess material.

In a separate fabrication process, the counterpart collector was coated over a length of 20 mm with a bilayered ELR/TPU construct. First, an initial ELR fiber layer was deposited by electrospinning (22G/17G, 0.37 mL h^−1^, 10 kV, 14 cm, 50 rpm, 3 min). Next, TPU and click‐ELR solutions were dual electrospun onto the rotating collector (18G, 1.7 mL h^−1^, 12 kV, 14 cm, 50 rpm, 5 min). Finally, TPU fibers alone were electrospun (18G, 2.5 mL h^−1^, 10 kV, 12 cm, 50 rpm, 5 min). Bridging fibers were removed manually before the counterpart and leaflet collectors were assembled and inserted into a 20 mm long segment of a self‐expanding closed‐cell nitinol stent (Sinus‐Repo Visual 6F, optimed Medizinische Instrumente, Germany). The collector‐stent assembly was then covered with TPU fibers by spinning for 5 min at a distance of 8.5 cm, followed by 7 min at 12 cm (18G, 3.5 mL h^−1^, 10 kV, 50 rpm), resulting in full embedding of the stent struts. During this process, the mandrel was moved in an alternating translational motion at a speed of 10 mm s^−1^ for 5 min, covering the collector assembly over a length of 40 mm. Additionally, an abluminal non‐porous layer was applied to the ELR/TPU valves by wet‐spinning TPU onto the collector‐stent assembly for 5 min by reducing the tip‐to‐collector distance to 8 cm (18G, 10 kV, 50 rpm, axial motion of 10 mm s^−1^). Before releasing the valve, the assembly was dried at 37°C for 24 h, followed by incubation in PBS at 37°C for an additional 24 h.

### 4.5 In Vitro Hydrodynamic Evaluation

An in‐house developed mock circulatory system was used to characterize the functionality of the venous valve under the following flow and pressure conditions: 60 mmHg proximal pressure, 500 to 1000 mL min^−1^ flow rates, and 60 bpm. The applied proximal pressure approximately corresponds to the hydrostatic venous pressure at knee level in the standing position [88, 89], and the tested flow rates were chosen to lie within the range of common femoral vein flow reported in vivo [90, 91, 92]. Clamp‐on ultrasound flowmeter (CO.55/080 V2.0, SONOTEC, Germany) was used to monitor the flow and pressure transducers (Xtrans, CODAN, Germany) were placed at the inflow and outflow sides of the valve. The device was monitored and actuated using an application built with LabVIEW 2023 (National Instruments, Germany). The communication between software and peripherals was achieved with a NI USB‐9600 data acquisition system (National Instruments, Germany). The valve cycles were visually observed from the outflow side and recorded (1080p at 60 fps; iPhone 13 Pro, Apple, USA) to evaluate leaflets’ movement and closing behavior. The pressure and flow recorded over 10 consecutive cycles were used to calculate the Effective Orifice Area (EOA), the regurgitant volume, the regurgitant fraction, and the mean transvalvular pressure drop according to the DIN EN ISO 5840–1 standard for heart valves [60], as there are no ISO standards for venous valves. The regurgitant fraction was calculated as the ratio of regurgitant volume to the forward flow volume, expressed as a percentage. The EOA is a standard parameter for the functionality assessment of valve substitutes calculated according to Equation 1 in cm^2^:

$$EOA=\frac{q_{RMS}}{51,6\sqrt{\frac{\Delta P_{mean}}{\rho }}}$$

where q_RMS_ is the root mean square of the forward flow during the positive differential pressure period in mL s^−1^, ΔP_mean_ is the corresponding mean pressure difference (MPD) in mmHg, and ρ is the density of the test fluid in g cm^−3^ (for water 1 g cm^−3^). The EOA was also calculated as a percentage of the test tube's lumen (0.7854 cm^2^ for a 10 mm inner diameter test tube).

### 4.6 PVV Delivery Simulation

Valves were crimped to a minimum diameter of 2.8 mm (THV Crimper 9600CR, Edwards, USA). The crimped valve was cooled to 4°C using iced water and was pushed into a mock delivery system consisting of a PTFE tube (PTFE Tube Shop, Netherlands) with an inner diameter of 3.2 mm and an outer diameter of 4 mm (12 Fr). Afterward, the valve was deployed into a silicone tube (inner diameter 10 mm) in a water bath at 37°C by advancing it until release to simulate a delivery procedure.

### 4.7 Biaxial Tensile Testing

Single‐material TPU constructs (15 mm × 15 mm, n = 3, thickness 131.7 ± 12.6 µm) and trilayered ELR/TPU constructs (15 mm × 15 mm, n = 3, 146.7 ± 22.6 µm) were tested using a BioTester 3000 (CellScale, Canada). The samples were produced by electrospinning onto a cylindrical collector with a diameter of 10 mm rotating at 50 rpm as during valve production, and subsequently cut into squares. Sample thickness was measured using a digital thickness gauge (Mitutoyo, Japan), and specimens were mounted on the hooks of the sample holder, resulting in an effective test area of 10 × 10 mm. Tensile strain was applied at a rate of 0.1 mm s^−^^1^, and forces in the x‐ and y‐directions (i.e., axial and circumferential) were recorded continuously using a 5 N load cell. Tensile moduli in the axial and circumferential directions were calculated from the slope of the stress–strain curves in the 0%–5% and 20%–40% strain ranges using a custom MATLAB script (MathWorks, USA).

### 4.8 Burst Strength Measurement

Planar trilayered ELR/TPU samples (15 mm × 15 mm, n = 3, thickness 147.7 ± 6.7 µm) were mounted in a testing chamber containing a 4 mm diameter opening and exposed to increasing pressure applied with a syringe pump until material failure occurred. The pressure at failure was recorded as the burst pressure. Pressure was monitored using a pressure sensor (MIDAS 401001/000, Jumo, Germany) connected to a NI USB‐9600 data acquisition system and controlled with custom Python software (Python Software Foundation, USA).

### 4.9 Scanning Electron Microscopy

SEM was used for the evaluation of the delamination test, cross‐sectional analysis of the electrospun constructs, the platelet adhesion assay, and microstructural examination of the valve after delivery simulation. Samples were sputter‐coated with gold (7 nm layer; SCD 005, BAL‐TEC, Germany). Imaging was performed with a scanning electron microscope (JSM‐6390, JEOL, Germany) operated at an accelerating voltage of 10 kV.

### 4.10 Delamination Test

Disks of bilayered ELR/TPU constructs (n = 3, thickness 127.3 ± 14.2 µm) with a diameter of 5 mm were mounted in the sample holder of an in‐house developed flow chamber. The parallel plate flow chamber had a channel height of 300 µm and a width of 12 mm. The samples were positioned at the center of the channel to ensure laminar flow over the width of the ELR covered surface. A compliance chamber was connected between the chamber inlet and the peristaltic pump (Ismatec Laboratoriumstechnik, Germany). The entire system was filled with distilled water. The samples were exposed to a shear stress of 30 dynes cm^−2^ for 1 h at room temperature. Afterward, the samples were air‐dried and examined by SEM.

### 4.11 Cross‐Section Visualization

To visualize the cross‐sectional architecture of the bi‐ and trilayered ELR/TPU constructs as well as TPU membranes with non‐porous and porous regions, samples were processed into 5 µm slices by standard histological sectioning. Slices were mounted with mounting medium (Dako, Agilent Technologies, USA), covered with glass coverslips, and imaged either by bright field microscopy (BZ‐X800, Keyence, Japan) or, when applicable, by fluorescence microscopy exploiting the autofluorescence of ELRs with the GFP filter (OP‐87763, BZ‐X Filter GFP, Keyence, Japan). Cross‐sections of the materials were also imaged by SEM.

### 4.12 Blood Drawing

Blood from healthy volunteers was collected by phlebotomy using the S‐Monovette Citrate 9NC 0.106 mol l^−1^ 3.2% (Sarstedt, Germany) following a protocol approved by the TUM Ethics Committee (votum number 2023‐40‐S‐SR). Platelet‐poor plasma (PPP) was prepared from citrate‐enriched whole blood by centrifugation at 2000 g for 15 min; to obtain platelet‐rich plasma (PRP), the citrate‐enriched blood was centrifuged at 180 g for 5 min. The platelet concentration of PRP was determined using a blood analyzer (pocH‐100i, Sysmex, Germany).

### 4.13 Platelet Adhesion Assay

Bilayered ELR/TPU constructs were cut into 5 × 5 mm pieces and washed in 70% ethanol two times for 1 h under gentle shaking, followed by sterile PBS washing three times for 15 min. The samples (n = 5) and ePTFE (GORE‐TEX Cardiovascular Patch, W. L. Gore & Associates, USA) as reference material were placed in a 48‐well plate (VWR, USA). Fibrin gel served as the positive control and was prepared by mixing 1:1 thrombin and fibrinogen mixtures in tris‐buffered saline solution (TBS) and polymerized at 37°C for 45 min. The TBS solution consisted of 137 mM sodium chloride (Sigma‐Aldrich, Germany), 2.7 mM potassium chloride (Merck, Darmstadt, Germany), 5 mM tris base (Sigma‐Aldrich, Germany), and 27 mM tris hydrochloride (Sigma‐Aldrich, Germany) in deionized (DI) water and the pH was set to 7.4 using hydrochloric acid (Sigma‐Aldrich, Germany). The thrombin mixture consisted of 6 U mL^−1^ thrombin (Sigma‐Aldrich, Germany) and 7.5 mM calcium chloride (Sigma‐Aldrich, Germany) in TBS. The fibrinogen mixture consisted of 10 mg mL^−1^ fibrinogen from human plasma (Millipore Sigma, Merck KGaA, Germany) in TBS.

PRP platelet concentration was measured (pocH‐100i, Sysmex, Germany) and adjusted to a concentration of 3 × 10^5^ cells µL^−1^ by dilution with PPP. 400 µL PRP with an adjusted platelet concentration were then pipetted into each well containing the samples and the fibrin gel. After an incubation time of 1 h at 37°C and 5% CO_2_, the samples were washed in PBS for 5 min, fixated for 10 min in 2% glutaraldehyde in PHEM buffer (Sigma‐Aldrich, Germany), and then washed in PHEM buffer three times for 5 min. The PHEM buffer was prepared by dissolving 60 mM piperazine‐1,4‐bis(2‐ethanesulfonic acid) (PIPES), 25 mM 2‐[4‐(2‐hydroxyethyl)piperazin‐1‐yl] ethane sulfonic acid (HEPES), 10 mM ethylene glycol‐ bis(β‐aminoethyl)‐N,N,Nʹ,Nʹ‐tetraacetic acid (EGTA), and 2 mM magnesium chloride hexahydrate (MgCl2 · 6 H2O) in DI water. The pH was set to 6.9 using sodium hydroxide (Sigma‐Aldrich, Germany). They were then dehydrated by a series of increasing concentrations of ethanol (30, 50, 70, 80, 90 and 3 × 100%) prepared in PHEM buffer each for 10 min at room temperature and subsequent critical point drying (CPD7501, Quorum, USA) using carbon dioxide at 33°C and 73 bar. The samples were imaged by SEM and the surface area covered by platelets was determined. For each of the five independent samples, one image segment of an approximate size of 43 µm × 32 µm was evaluated using ImageJ 1.53e (U.S. National Institutes of Health, USA).

### 4.14 Hemolysis

Trilayered ELR/TPU constructs were cut into 10 × 10 mm pieces and washed in 70% ethanol twice for 1 h under gentle shaking, followed by sterile PBS washing three times for 15 min. Prior to incubation with the samples, 200 µL citrate‐enriched whole blood were diluted in 10 mL 0.9% saline solution (B. Braun, Germany). The samples (n = 5), high density PE (Raumedic, Germany), and ePTFE (GORE‐TEX Cardiovascular Patch, W. L. Gore & Associates, USA) as reference materials were placed in conical 1.5 mL tubes (Eppendorf, Germany) and 700 µL citrate‐enriched diluted whole blood were added to each tube. For the controls, citrate‐enriched whole blood diluted in deionized water (200 µL whole blood in 10 mL DI water) was used as the positive control (PC), and citrate‐enriched whole blood diluted in saline solution (200 µL whole blood in 10 mL 0.9% saline) as the negative control (NC). The control solutions were transferred into empty 1.5 mL conical tubes. After an incubation time of 2 h at 37°C under shaking at 100 rpm, the tubes containing the sample and the controls were centrifuged at 1000 g for 10 min. Next, the supernatant was transferred to a 96‐well plate (100 µL per well) (VWR, USA) to measure absorbance values at 545 nm with a spectrophotometer (Spark, Tecan, Switzerland). Hemolysis rate was calculated using the following formula according to Equation 2:

$$\%\;\mathrm{hemolysis}\;=\frac{A_{S}-A_{NC}}{A_{PC}-\;A_{NC}}\times 100$$

where AS, ANC, and APC denote the absorbance of the sample, the negative control, and the positive control at 545 nm, respectively.

### 4.15 Cell Isolation

Primary human umbilical vein endothelial cells (HUVECs) and primary human umbilical vein smooth muscle cells (HUVSMCs) were isolated from fresh human umbilical cords as previously described [93]. The tissue was provided by the Tissue Bank of the University Hospital of the Technical University of Munich (MTBIO), in accordance with its established regulations and with prior approval from the TUM Ethics Committee (votum number 2023‐531‐S‐KH). HUVECs were seeded on 2% gelatin (Sigma‐Aldrich, Germany) pre‐coated cell culture flasks (VWR, USA) and cultured with endothelial cell growth medium (EBM‐2, Lonza, USA) supplemented with ECM‐2 kit (0.1% insulin, 0.1% gentamicin, 0.1% ascorbic acid, 0.4% human fibroblast growth factor, 0.1% endothelial growth factor, 0.04% hydrocortisone, 0.1% epidermal growth factor, 0.1% heparin (Lonza, USA)), and 1% antibiotic/antimycotic solution (Gibco, USA). Cells were incubated in 5% CO_2_ and 95% humidity at 37°C and serially passaged, when necessary, with regular medium changes every 2 to 3 days. HUVSMCs were cultured in high glucose Dulbecco's Modified Eagle Medium (DMEM) (Gibco, USA), supplemented with 10% fetal bovine serum (Gibco, USA) and 1% antibiotic‐antimycotic (Gibco, USA) at 37°C in a humidified atmosphere containing 5% CO_2_ and serially passaged, when necessary, with regular medium changes every 2 to 3 days.

### 4.16 In Vitro Endothelialization

HUVECs in passage 3 were used for this experiment. The samples (bilayered ELR/TPU constructs and TPU membranes; n = 3 for each material) were cut into 6 mm discs, washed in 70% ethanol twice for 1 h under gentle shaking, and in sterile PBS three times for 15 min. The discs were placed in 96‐well plates (ELR/TPU discs were oriented with the ELR layer facing upward) and 100 µL of HUVEC suspension containing 5 × 10^5^ cells mL^−1^ was seeded on each disc and incubated at 37°C in 5% CO_2_ and 95% humidity. HUVECs seeded on 2% gelatin pre‐coated wells served as positive control. After 24 and 72 h, the medium was removed, and samples were fixed by adding 100 µL of 4% formaldehyde (Carl Roth, Germany) in PBS for 40 min with subsequent PBS washing three times 15 min and stored at 4°C until further processing for immunostaining.

### 4.17 In Vitro Smooth Muscle Cell Infiltration

HUVSMCs in passage 3 were used for this experiment. The samples (bi‐ and trilayered ELR/TPU constructs, porous TPU, and TPU samples featuring a non‐porous region; n = 3 per time point) were mounted in in‐house designed sample holders with a diameter of 11 mm, which were additively fabricated by vat photopolymerization (BioMed Clear resin, Form3, Formlabs, USA). Then, the mounted samples were washed twice in 70% ethanol for 1 h, rinsed in PBS for three times 1 h, and then dried under the flow hood for 1 h. HUVSMCs were seeded onto the samples at a density of 10^4^ cells cm^−2^. After incubation for 1 h at 37°C to allow for cell adhesion, 4 mL culture medium were added to the samples in the wells. The cell‐seeded samples were cultured for up to 28 days with medium changes every 3 days. At day 7 and 28, samples were rinsed in PBS, fixed in 4% formaldehyde for 30 min, washed in PBS for three times 15 min, and stored at 4°C until further processing for immunostaining.

### 4.18 Immunostaining

Immunofluorescence staining was performed to evaluate in vitro endothelialization. First, a solution containing 5% normal goat serum (Dako, Denmark) and 0.1% Triton X‐100 (Sigma Aldrich, Germany) in PBS was added to the sample slices for blocking of unspecific binding sites and permeabilization of the cell membrane. The samples were incubated for 1 h with anti‐CD31 mouse monoclonal antibody (Sigma Aldrich, Germany) diluted 1:100. Next, the samples were incubated for 1 h with a 1:400 dilution of a fluorescein‐conjugated AlexaFluor 594 anti‐mouse secondary antibody produced in goat (Life Technologies, Germany). Both the primary and secondary antibodies were diluted in a diluent PBS solution containing 1% bovine serum albumin (Sigma Aldrich, Germany) and 0.1% sodium azide (Sigma Aldrich, Germany). HUVECs in well plates incubated without the primary antibody were used as negative controls. For visualization of the nucleus, the samples were counterstained with 4′,6‐diamidino‐2‐phenylindole (DAPI) nucleic acid stain (Invitrogen, USA) at a concentration of 0.2 µg mL^−1^ for 5 min at room temperature. Before imaging, the samples were carefully removed from the well plate and placed on a microscope slide (Epredia, Netherlands). Images were acquired using the fluorescence microscope equipped with DAPI filter (OP‐87762, BZ‐X Filter DAPI, Keyence, Japan) and TexasRed filter (OP‐87765, BZ‐X Filter TexasRed, Keyence, Japan).

For the evaluation of in vitro HUVSMC infiltration, samples were cut into 5 µm slices by standard histological sectioning. Nuclei were counterstained with DAPI at 0.2 µg mL^−^^1^ under gentle shaking. After 5 min of incubation, the sample slices were washed three times in PBS, mounted with mounting medium, covered with glass coverslips, and imaged by the fluorescence microscope equipped with a DAPI filter.

### 4.19 Statistics

Statistical analyses were conducted using Prism 10.5.0 (GraphPad Software, USA). Normality was assessed with the Shapiro–Wilk test. For normally distributed data, unpaired t‐tests with Welch's correction were applied. For non‐normally distributed data, the Mann–Whitney test was used. A *p*‐value of *p* ≤ 0.05 was chosen as a threshold for significance.

## Conflicts of Interest

The authors declare no conflicts of interest.

## Supporting information




**Supporting File**: adhm70918‐sup‐0001‐SuppMat.docx.

## Data Availability

The data that support the findings of this study are available from the corresponding author upon reasonable request.
