# Supplementary material for: A Versatile Engineering Platform for the Fabrication of Prosthetic Venous Valves Using Electrospinning
Source: Adv Healthc Mater. 2026 Feb 9;15(16):e04851. doi: 10.1002/adhm.202504851 (PMC13107922; doi:10.1002/adhm.202504851)
Supplement: Supplementary file 1 — Supporting File: adhm70918‐sup‐0001‐SuppMat.docx. [file ADHM-15-0-s001.docx]

Supporting Information

A Versatile Engineering Platform for the Fabrication of Prosthetic Venous Valves Using Electrospinning

Dario Arcuti, Salma Mansi, Dominic Biebl, Malin Reuter, Maximilian Grab, José Carlos Rodríguez-Cabello and Petra Mela*

D. Arcuti, S. Mansi, D. Biebl, M. Reuter, M. Grab, P. Mela,

Technical University of Munich, Germany; TUM School of Engineering and Design, Department of Mechanical Engineering, Chair of Medical Materials and Implants; Munich Institute of Biomedical Engineering; Munich Institute of Integrated Materials, Energy and Process Engineering. Boltzmannstraße 15, 85748 Garching, Germany
E-mail: petra.mela@tum.de

M. Grab

Department of Cardiac Surgery, LMU University Hospital, Ludwig Maximilian University Munich, Munich, Germany.

M. Grab

Deutsches Zentrum für Herz-Kreislauf-Forschung (German Centre for Cardiovascular Research), Partner Site Munich Heart Alliance, Munich, Germany.

J.C. Rodríguez-Cabello

Bioforge Lab (Group for Advanced Materials and Nanobiotechnology), Laboratory for Disruptive Interdisciplinary Science (LaDIS), CIBER-BBN, Edificio LUCIA, Universidad de Valladolid, Valladolid, Spain


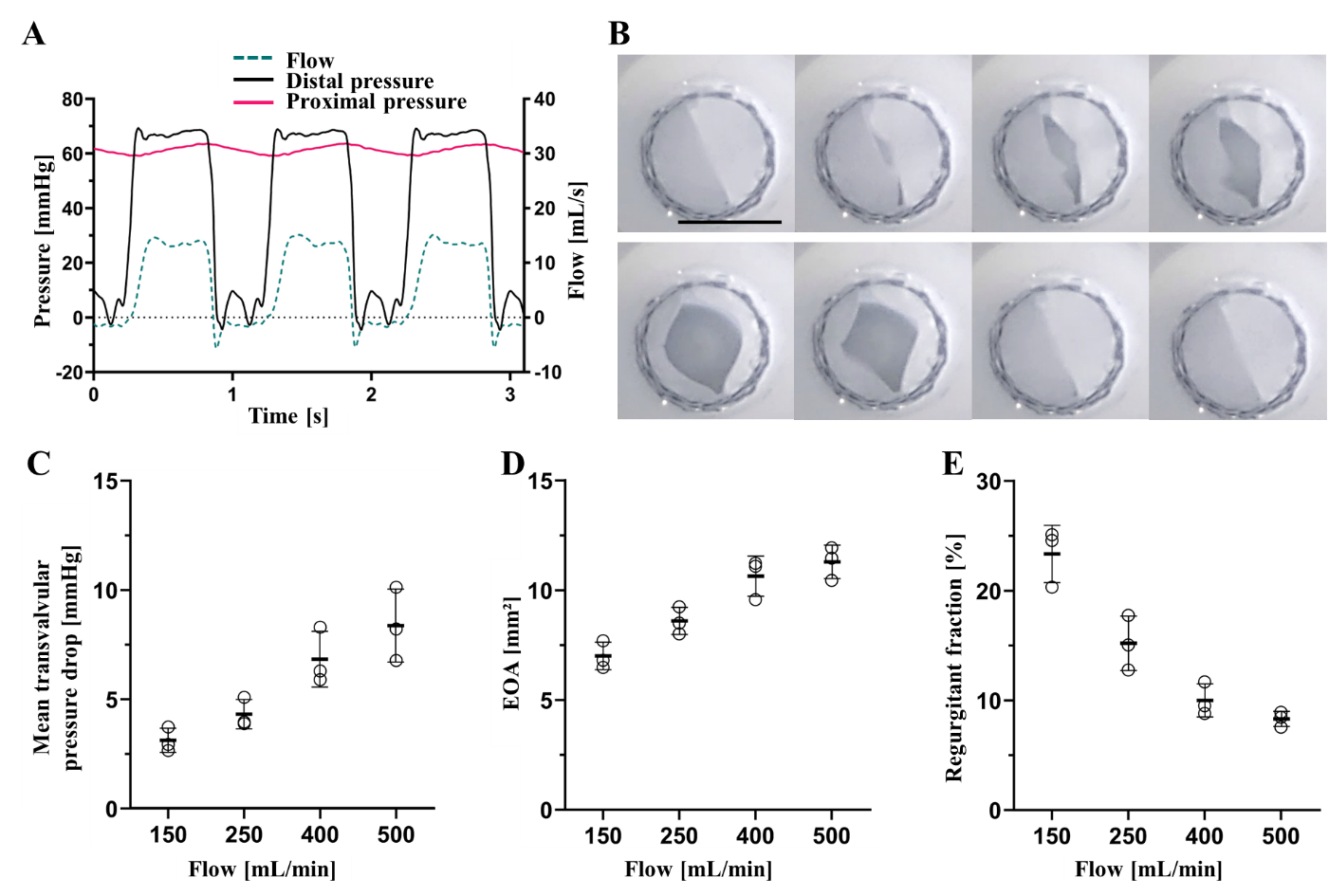


**Figure S1**. Hydrodynamic evaluation of 6 mm single-material TPU bicuspid valves in a mock circulatory system. A) Typical pressure and flow profiles and B) still frames of an opening and closing cycle of a valve at a flow rate of 400 mL min^-1^. C) Mean transvalvular pressure drop, D) effective orifice area, and E) regurgitant fraction. Data represented as mean values (n = 3) ± standard deviation and as individual values. Scale bar 5 mm

*
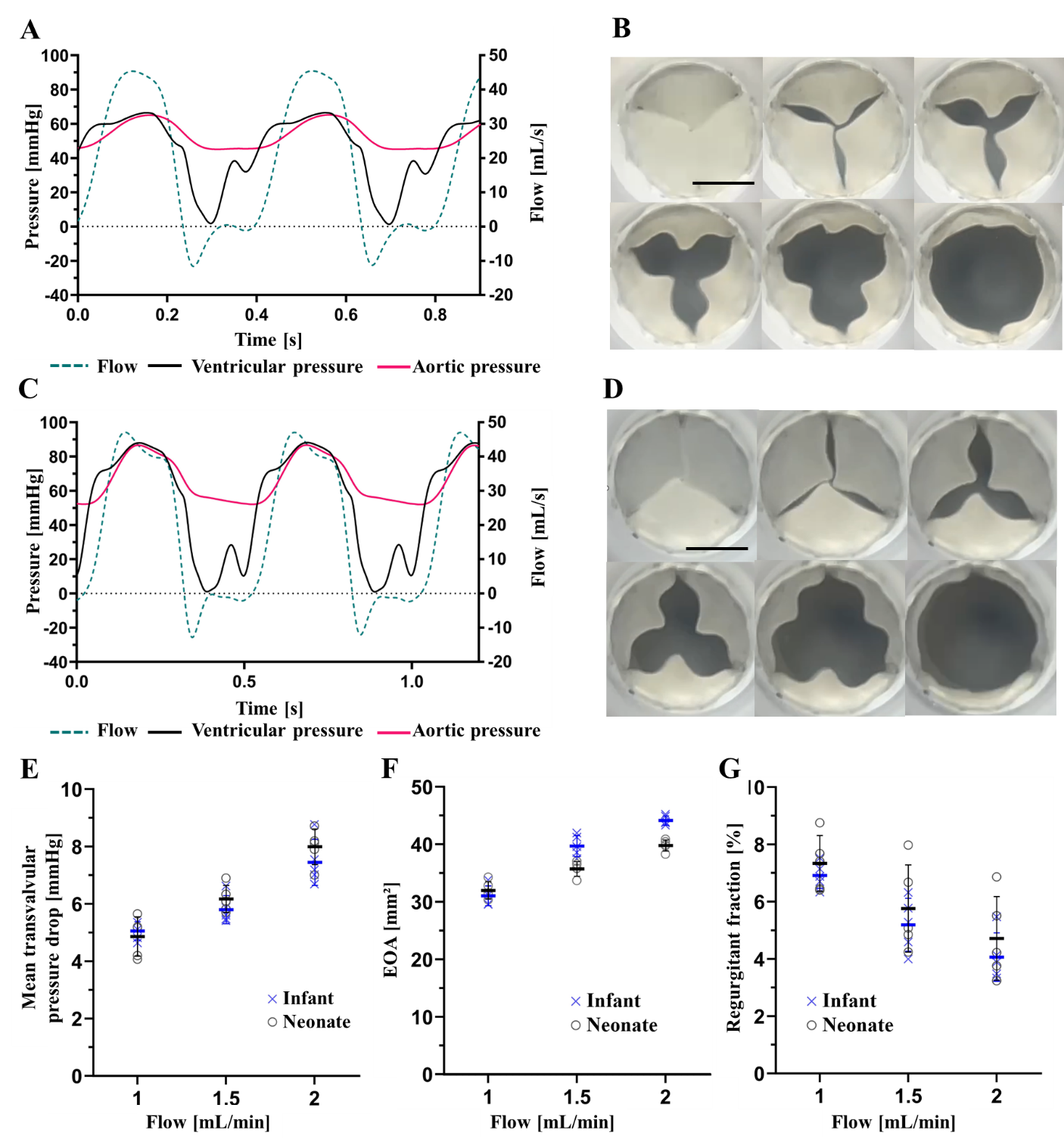
*

**Figure S2**. Hydrodynamic evaluation of 10 mm single-material TPU tricuspid valves in a mock circulatory system under pediatric aortic conditions. A) Typical pressure and flow profiles and B) still frames of an opening and closing cycle under neonatal aortic conditions (65/45 mmHg, 150 bpm) at a flow rate of 1000 mL min^-1^. C) Typical pressure and flow profiles and D) still frames of an opening and closing cycle under infant aortic conditions (88/50 mmHg, 120 bpm) at a flow rate of 1000 mL min^-1^. E) Mean transvalvular pressure drop, F) EOA, and G) regurgitant fraction. Data represented as mean values (n = 5) ± standard deviation and as individual values. Scale bars 5 mm


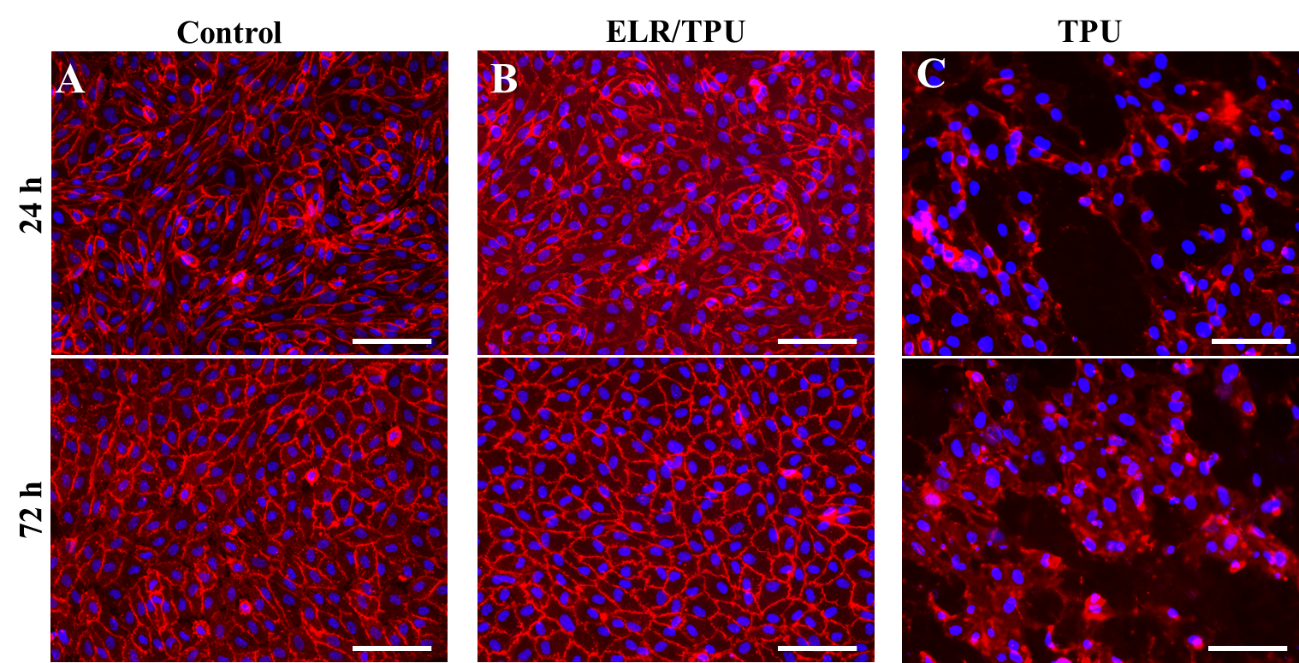


**Figure S3.** Representative fluorescence images of endothelialized surfaces showing CD31 staining (red) and DAPI nuclear counterstaining (blue). A confluent endothelial cell layer was observed on A) gelatin-coated wells (positive control) and B) ELR/TPU constructs after 24 h and 72 h of culture, whereas C) TPU samples showed incomplete coverage at both time points. Scale bars 100 µm


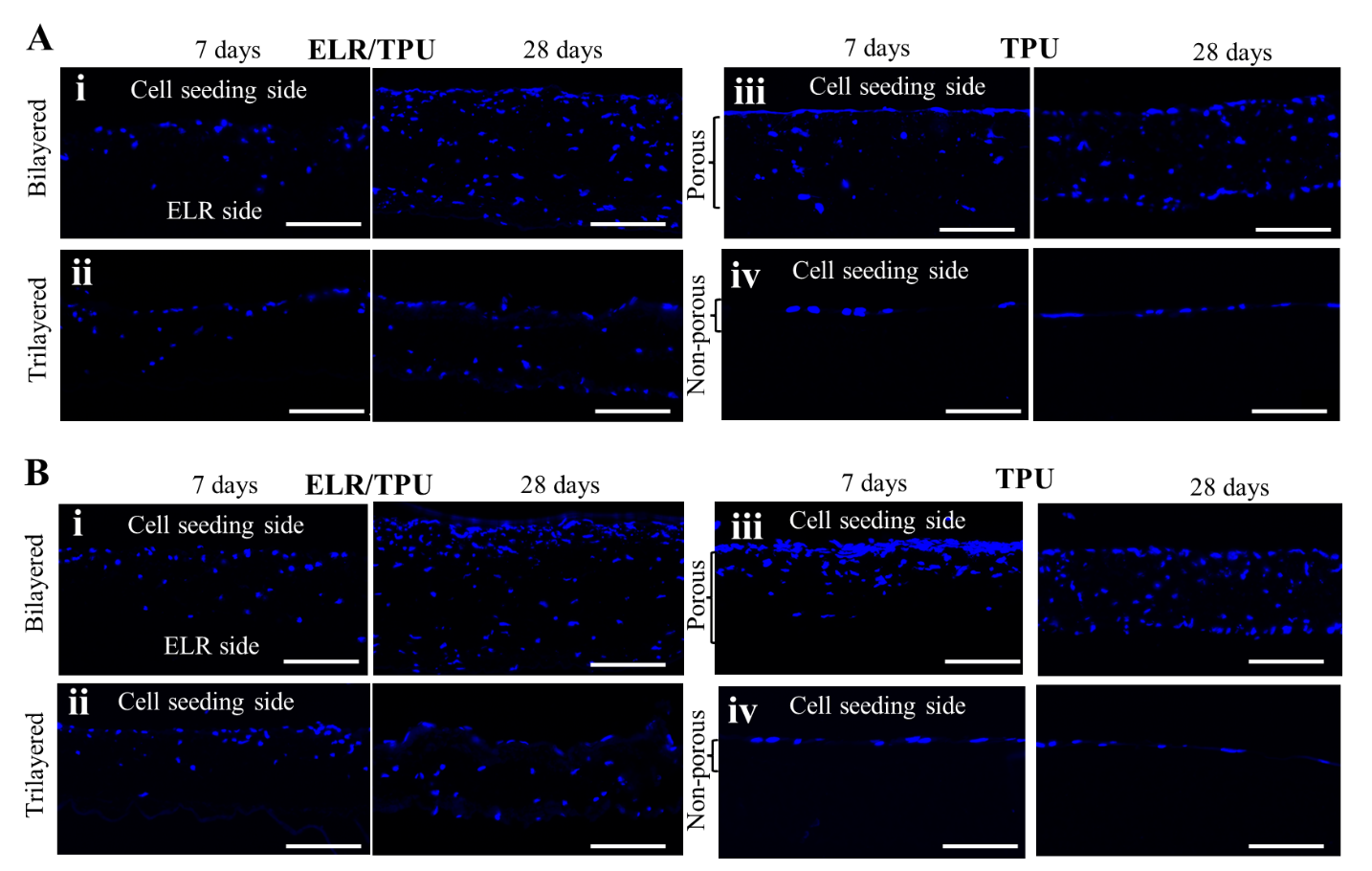


**Figure S4.** Fluorescence images of cross-sectional cuts of additional replicates (A and B) used to assess SMC infiltration with DAPI nuclear counterstain (blue). Scale bars 100 µm


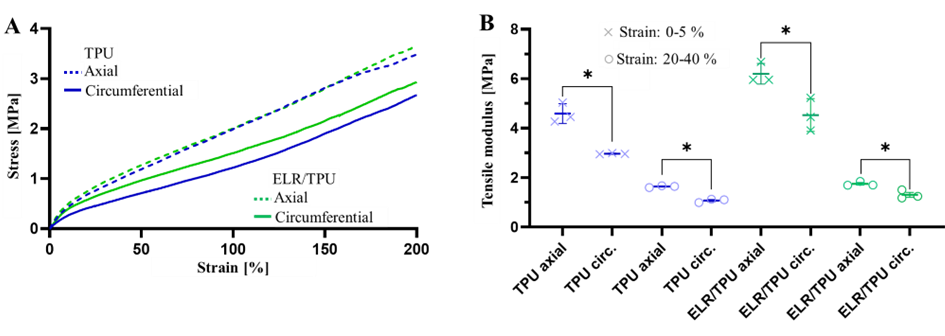


**Figure S5:** Mechanical properties of single and multi-material electrospun constructs. A) Representative biaxial stress–strain curves in the circumferential (solid lines) and axial direction (dashed lines) of a single-material TPU (blue) and trilayered ELR/TPU (green) construct up to 200 % strain. B) Tensile moduli calculated from the slope of the stress–strain curves in the 0–5 % and 20–40 % strain ranges. Data represented as mean values (n = 3) ± standard deviation and as individual values. Asterisks (*) mark statistically significant differences based on a p-value of 0.05.


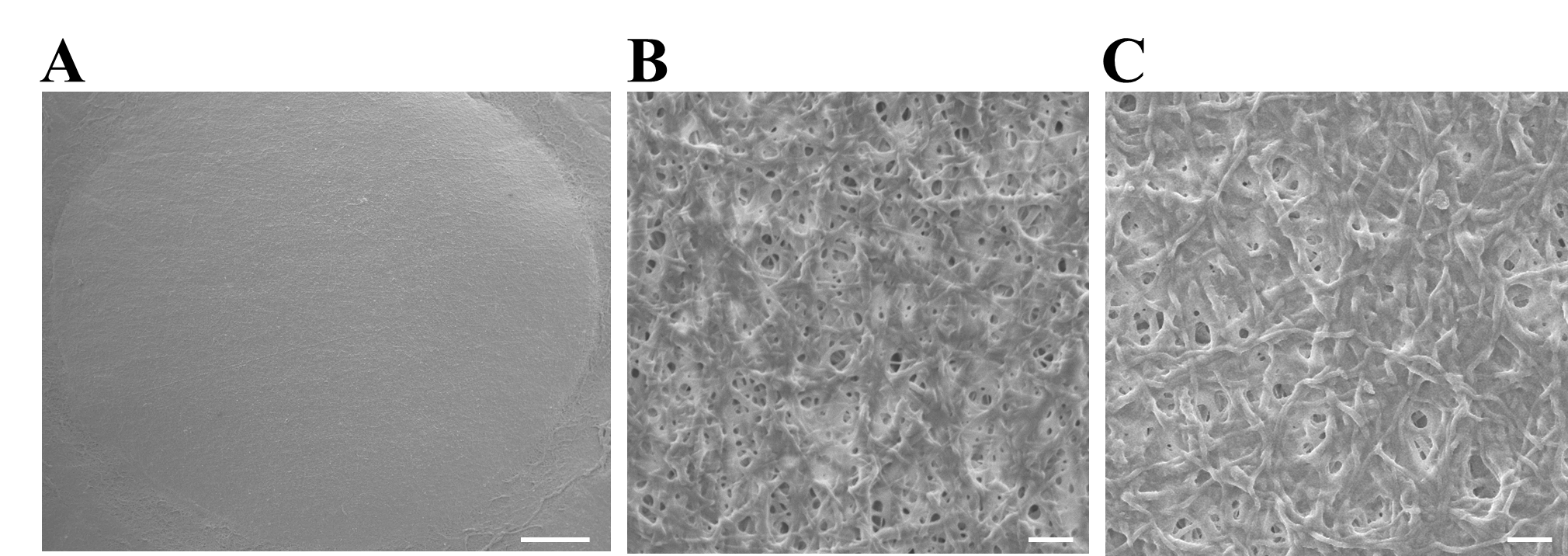


**Figure S6.** A) View of the whole ELR area exposed to shear stress. B) Close up image of ELR covered surface. C) Unsheared ELR control surface. Scale bars: A: 500 μm, B-C: 10 μm


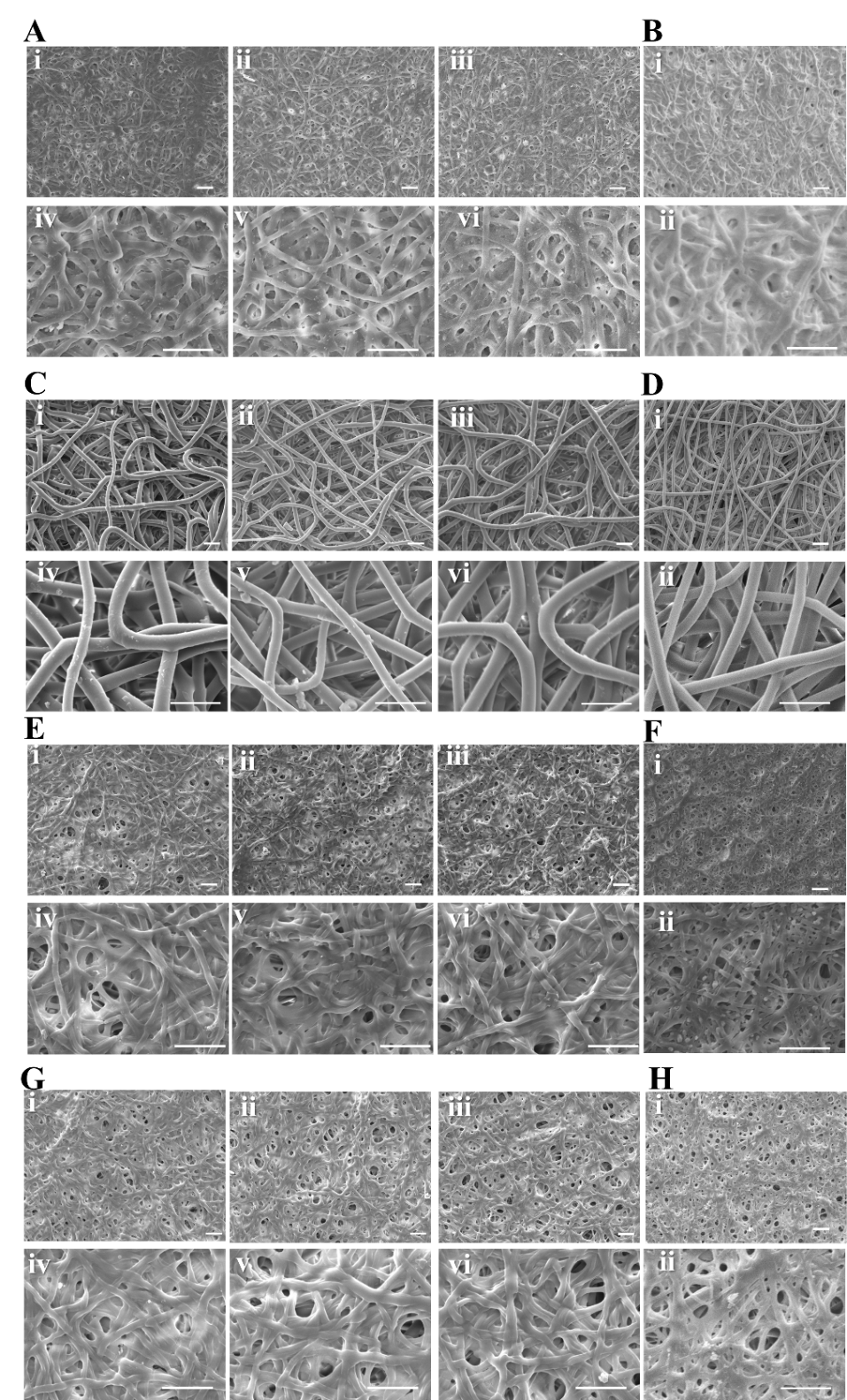


**Figure S7.** Effect of crimping on fiber morphology. Representative images of valve surfaces after crimping and simulated delivery (A, C, E, G) and uncrimped controls (B, D, F, H) using SEM. Overview and zoomed-in images of the A,B) luminal surface and C,D) abluminal surface of the stent cover, as well as E,F) luminal surface and G,H) parietal surface of the leaflet. Scale bars 10 μm


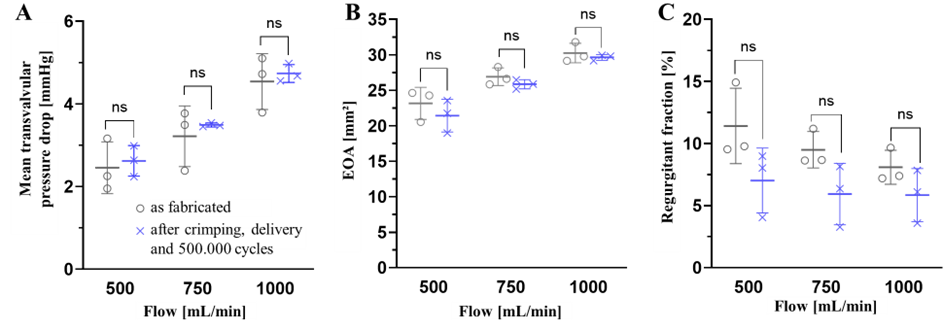


**Figure S8**. Hydrodynamic evaluation of 10 mm bicuspid TPU valves as fabricated and after crimping, simulated delivery and 500,000 cycles in a mock circulatory system at a proximal pressure of 60 mmHg. A) Mean transvalvular pressure drop, B) effective orifice area, and C) regurgitant fraction. Data represented as mean values (n = 3) ± standard deviation and as individual values. “ns” indicates non-significant differences.
